# Brain structure and connectivity in psoriasis and associations with depression and inflammation; findings from the UK biobank

**DOI:** 10.1016/j.bbih.2022.100565

**Published:** 2022-11-21

**Authors:** Georgia Lada, Peter S. Talbot, Hector Chinoy, Richard B. Warren, Martyn McFarquhar, C. Elise Kleyn

**Affiliations:** a Dermatology Centre, Salford Royal NHS Foundation Trust, National Institute for Health Research Manchester Biomedical Research Centre, The University of Manchester, Manchester, M13 9PL, UK; b Division of Neuroscience and Experimental Psychology, Faculty of Biology, Medicine and Health, The University of Manchester, Manchester, M13 9PL, UK; c National Institute for Health Research Manchester Biomedical Research Centre, Manchester University NHS Foundation Trust, The University of Manchester, Manchester, M13 9PL, UK

**Keywords:** Brain, Neuroimaging, Psoriasis, Depression, Psoriatic arthritis, Inflammation, UK Biobank, Suicidality

## Abstract

**Background:**

Psoriasis is a chronic systemic inflammatory skin disease, coexisting with depression in up to 25% of patients. Little is known about the drivers of comorbidity, including shared neurobiology and depression brain imaging patterns in patients. An immune-mediated crosstalk between the brain and skin has been hypothesized in psoriasis. With the aim of investigating brain structure and connectivity in psoriasis in relation to depression comorbidity, we conducted a brain imaging study including the largest psoriasis patient sample to date (to our knowledge) and the first to investigate the role of depression and systemic inflammation in brain measures. Effects of coexisting psoriatic arthritis (PsA), which represents joint involvement in psoriasis and a higher putative inflammatory state, were further explored.

**Methods:**

Brain magnetic resonance imaging (MRI) data of 1,048 UK Biobank participants were used (131 comorbid patients with psoriasis and depression, age-and sex-matched to: 131 non-depressed psoriasis patients; 393 depressed controls; and 393 non-depressed controls). Interaction effects of psoriasis and depression on volume, thickness and surface of a-priori defined regions of interest (ROIs), white matter tracts and 55x55 partial correlation resting-state connectivity matrices were investigated using general linear models. Linear regression was employed to test associations of brain measures with C-reactive protein (CRP) and neutrophil counts.

**Results:**

No differences in regional or global brain volumes or white matter integrity were found in patients with psoriasis compared to controls without psoriasis or PsA. Thickness in right precuneus was increased in psoriasis patients compared to controls, only when depression was present (β = 0.26, 95% CI [Confidence Intervals] 0.08, 0.44; p = 0.02). In further analysis, psoriasis patients who had PsA exhibited fronto-occipital decoupling in resting-state connectivity compared to patients without joint involvement (β = 0.39, 95% CI 0.13, 0.64; p = 0.005) and controls (β = 0.49, 95% CI 0.25, 0.74; p < 0.001), which was unrelated to depression comorbidity. Precuneus thickness and fronto-occipital connectivity were not predicted by CRP or neutrophil counts. Precuneus thickening among depressed psoriasis patients showed a marginal correlation with recurrent lifetime suicidality.

**Conclusions:**

Our findings provide evidence for a combined effect of psoriasis and depression on the precuneus, which is not directly linked to systemic inflammation, and may relate to suicidality or altered somatosensory processing. The use of the UK Biobank may limit generalizability of results in populations with severe disease.

## Highlights

• Psoriasis showed no effect on regional brain volumes or structural connectivity.
• Psoriasis and depression comorbidity was associated with right precuneus thickening.
• Psoriatic arthritis heightens resting-state frontooccipital decoupling in psoriasis.
• These findings were not predicted by baseline C-reactive protein or neutrophils.
• Suicidality may contribute to precuneus thickness in depressed psoriasis patients.

## 1 Introduction

Psoriasis is a chronic skin disease affecting ∼2% of the population. It commonly manifests as erythematous and scaly plaques over extensor surfaces and is regarded as a systemic inflammatory condition (Parisi et al., 2013). Nine to thirty percent of patients have joint involvement in the form of psoriatic arthritis (PsA), representing a subgroup with higher putative inflammatory burden and functional impairment (Ogdie et al., 2012; Scher et al., 2019).

Depression is overrepresented in psoriasis, worsening disability and disease outcomes (Koo et al., 2017). One in five patients meet the major depressive disorder (MDD) criteria (Kleyn et al., 2020); up to half of those with coexistent PsA have a depressive syndrome (Lada et al., 2022). The drivers and underlying neurobiology of the psoriasis-depression comorbidity are not clear. Whereas poor body-image and experience of itch and pain play a role, they cannot fully explain the bidirectional link between psoriasis and depression (Koo et al., 2017). Depression increases the risk of psoriasis in the general population (Chen et al., 2021) and the risk of PsA progression in psoriasis (Lewinson et al., 2017), independently of obesity and lifestyle, whilst antidepressants appear to protect against psoriasis (Tzeng et al., 2021). Notably, immunomodulating treatments benefit both the skin and mood in psoriasis (Griffiths et al., 2017). Furthermore, patients with psoriasis and other disorders with psychosomatic features exhibit high levels of alexithymia (marked by an inability to recognize and describe emotions) (De Berardis et al., 2007; Kleyn et al., 2020), which generally increases vulnerability to depression and suicidality, and has been associated with elevated serum C-reactive protein (CRP) among psychiatric patients (De Berardis et al., 2017, 2020). These observations, combined with evidence of brain and peripheral inflammation in MDD (Enache et al., 2019), gave rise to the hypothesis of systemic and neuro-immunological processes underlying the psoriasis-depression comorbidity (Hunter et al., 2016; Koo et al., 2017).

It is plausible that such processes along the “brain-skin axis” (Hunter et al., 2013), either mutually triggered or a shared consequence of stress, contribute to changes in brain connectivity and long-term structural scarring in comorbid patients. The brain regulates immune responses in stress-triggered psoriasis via the sympathetic nervous system and hypothalamic-pituitary-adrenal (HPA) axis (Hunter et al., 2013). Inversely, humoral, neural and cellular cytokine signalling from peripheral sites on the brain (Capuron and Miller, 2011; D'Mello et al., 2009) may result in microglia activation, atrophy and disturbed neurotransmission (Maes et al., 2011). Effects of systemic inflammation on brain architecture are largely unexplored in psoriasis; although they are increasingly shown in healthy adults and patients with MDD (Marsland et al., 2015). In MDD, high serum CRP levels have been associated with anterior cingulate cortex (ACC) and entorhinal thinning (Green et al., 2021; Meier et al., 2016) and microstructural and functional connectivity changes in the precuneus, posterior cingulate cortex (PCC) and medial prefrontal cortex (Kitzbichler et al., 2021).

There is little evidence regarding the neurobiological substrates of psoriasis. A study examining dementia risk in 62 psoriasis patients found no abnormalities in global brain metrics (volume and fractional anisotropy (FA)) or hippocampus volume (Pezzolo et al., 2021). Nevertheless, structural brain changes and neuroinflammation in non-neurodegenerative disease are often localized (Enache et al., 2019; Wang et al., 2021; Wartolowska et al., 2012), and regional cortical metrics were not assessed in the dementia study. Limited brain connectivity data in psoriasis concern itch and disgust processing, with a recent pilot study showing increases in FA in several tracts and in mentally-induced itch neurocircuitry in a small non-depressed sample with psoriasis (n = 14); true resting-state data were not obtained (Najafi et al., 2020).

It is unclear how findings from these studies relate to the underlying psoriasis immunopathology or comorbid low mood. The only study to date measuring neuroinflammation in psoriasis found no evidence of activated microglia, using a translocator protein (TSPO) ligand (Hunter et al., 2016). However, the authors argue a lack of substantial systemic disease burden in this sample, as most serum inflammatory markers (including CRP and interleukin-6 (IL-6) levels) in patients were at similar levels to controls. Patients did not have depression or other comorbidity except PsA (Hunter et al., 2016). As depression is increasingly recognized as a heterogeneous disorder (Milaneschi et al., 2020), with brain abnormalities often being present at onset (Qiu et al., 2014), it is important to understand whether depression in psoriasis presents with a distinct neurobiological phenotype and whether this is consistent with inflammation.

Using the largest patient sample to date, we hypothesized that psoriasis has independent effects on grey matter, white matter (WM) integrity and resting state functional connectivity in key regions for depression and neuroinflammatory processes, correlating with patients’ systemic inflammatory load; and that the brains of depressed psoriasis patients may show pronounced and distinct structural and functional differences. To test these hypotheses, we first aimed to investigate structural (volume, thickness, surface, WM integrity) and functional brain changes in psoriasis, in relation to depression comorbidity. Secondarily, we tested whether: a) joint involvement in the form of PsA independently affects brain measures; b) peripheral inflammatory marker levels in psoriasis at baseline and at imaging are associated with observed brain differences (examining neutrophils along with CRP, owing to their central role in psoriasis (Rocha-Pereira et al., 2004)); and whether c) psoriasis duration and lifetime suicidality correlate with changes in brain measures in psoriasis, based on reports that suicidality may independently drive neuroinflammation in MDD (Holmes et al., 2018).

## 2 Methods

### 2.1 Participants

**Fig. 1:**
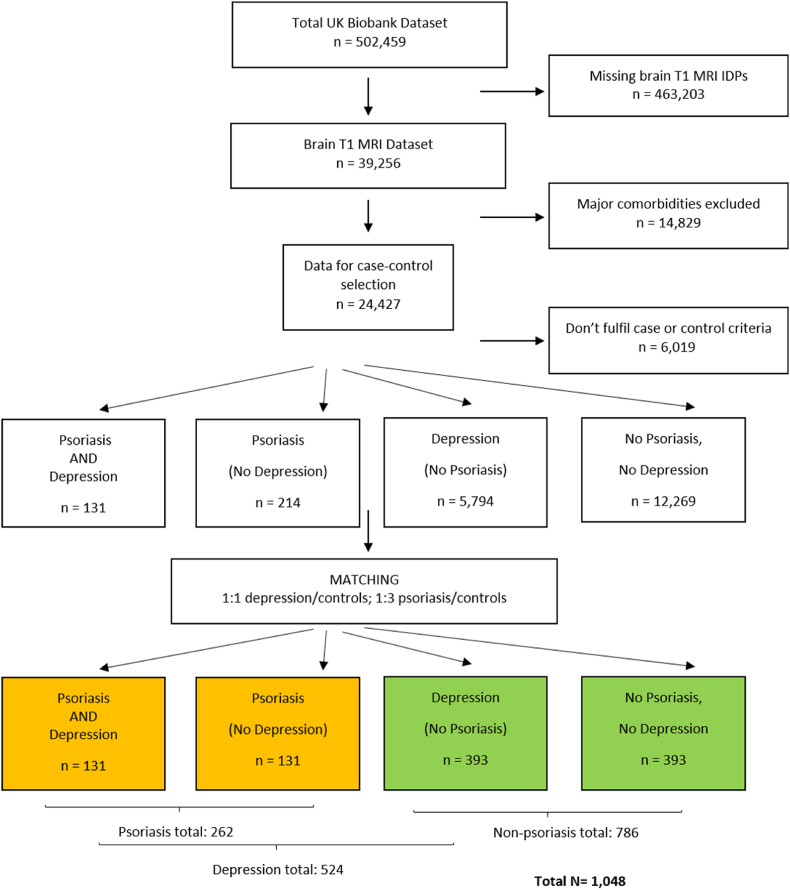
Flowchart. IDP = imaging-derived phenotypes.

The UK Biobank is a large prospective cohort study, enrolling participants aged between 40 and 69 years in multiple centres in the United Kingdom (UK) (Sudlow et al., 2015). A breadth of health data and biological measurements from participants is collected, with a subset undergoing brain Magnetic Resonance Imaging (MRI). In the September 2020 imaging data release, 39,256 UK Biobank participants in total had data for our T1 MRI-imaging derived phenotypes (IDPs) of interest. Out of those subjects, we excluded 14,829 participants with major comorbidities. Exclusion criteria included severe systemic, inflammatory, chronic infectious, and cardiometabolic disorders, atopic dermatitis, cancer history, central neurological and major psychiatric disorders (apart from depression), including schizophrenia, intellectual disabilities and dementia; for a comprehensive list see the Supplement, section A.1. 5,301 participants were further excluded for not fulfilling our psoriasis and depression case-control criteria (see subsections 2.2., 2.3., and the Supplement). The remaining participants (n = 18,408) were split into four groups, depending on psoriasis and depression presence (Fig. 1).

We observed differences among groups in sex and age, two major and complex neuroimaging confounders, as well as a low psoriasis-to-controls ratio. For these reasons, and because of the big dataset, where even small effects of confounders may lead to false-positive associations (Alfaro-Almagro et al., 2021; Smith and Nichols, 2018), we matched participants individually for sex and age (±1 years) (Fig. 1). We used a 1:3 ratio between the psoriasis and non-psoriasis groups and a 1:1 ratio between the depressed and non-depressed groups. Depressed psoriasis patients and depressed controls were further matched on depression phenotype (see 2.3. and Supplement). Where more than one matches were identified for a subject using the above (sex, age and depression phenotype) criteria, optimal matching was performed (Stuart, 2010) based on Body Mass Index (BMI) (the match with the closest BMI was selected), given that obesity increases psoriasis risk and affects brain volume, and is therefore expected to confound the investigated associations (Budu-Aggrey et al., 2019; Pan et al., 2021; Snekvik et al., 2017). Matching ratios were the maximum in order to retain in the analysis sample all subjects who were identified as comorbid before matching (n = 131 patients with both psoriasis and depression), as well as achieve acceptable covariate balance among the four groups with our criteria; it has been shown that increasing patient-control ratio above 1:3-1:4 does not significantly increase precision whilst it may increase bias through lowering the quality of matches (Austin, 2010; Linden and Samuels, 2013; Stuart, 2010).

After matching, 1,048 participants were entered in the T1 analysis (n = 262 with psoriasis; n = 786 without psoriasis); of those, 999 had diffusion-weighted MRI (dMRI) data (n = 252 with psoriasis) and 1,007 had resting state functional MRI (rs-fMRI) data (n = 254 with psoriasis). Based on these numbers, the study would have ∼90% power to detect effect sizes as small as Cohen's f = 0.10.

### 2.2 Psoriasis and psoriatic arthritis definitions

The UK Biobank sources of diagnostic data are: self-reported; hospital inpatient admission codes obtained through linkage; and linked primary care data available for approximately half of the cohort at the time of our data cut-off. To best balance misclassification bias and type II error given the prevalence of psoriasis, we used all participants who had a diagnosis for psoriasis in a primary care or hospital record by the time of imaging, applying an additional quality control for primary care records (see Supplement), as well as participants who had self-reported psoriasis by the time of imaging and also met at least one of the following criteria: a) had a diagnosis for psoriasis in primary care or hospital records of any date; b) had a PsA diagnosis in any source; c) had named a psoriasis-indicated treatment by the time of imaging. Controls did not have a psoriasis/PsA diagnosis (in any linked records or self-reported) and had not reported relevant treatments by the time of scan.

As a further validity check, we estimated the prevalence of identified cases at 1.8% of the total sample before matching, corresponding to the reported psoriasis prevalence in the UK (Parisi et al., 2013). To identify PsA cases among patients with psoriasis, we used PsA diagnosis in any source up to the date of the scan. Details about the diagnostic and treatment codes are presented in the Supplement.

### 2.3 Depression definition

In line with previous MDD research in the UK Biobank, lifetime depression was identified if participants met either the MDD threshold in the self-report Composite International Diagnostic Interview-Short Form (CIDI-SF; “CIDI” phenotype) (Kessler et al., 1998), which is part of the online Mental Health Questionnaire (MHQ) of the UK Biobank, or the probable MDD criteria defined by Smith et al. (“Smith” phenotype) by the time of imaging (Smith et al., 2013). Although the CIDI measure is based on a standardized tool, we and others have additionally used the “Smith” definition, as only a subset of participants (∼30% in our data release) completed the MHQ, which can result in power reduction and selection bias (Glanville et al., 2021; Shen et al., 2017). These definitions are detailed in the Supplement.

### 2.4 Magnetic resonance imaging (MRI) acquisition and measures

#### 2.4.1 T1-weighted MRI

We used imaging derived phenotypes (IDPs) of T1-weighted, diffusion and resting-state fMRI imaging data of the UK Biobank. MRI acquisition and pre-processing of images was performed by the UK Biobank according to the processing and quality control imaging pipeline, which has been described previously (Alfaro-Almagro et al., 2018) and can be found online, along with the standard imaging protocol (https://www.fmrib.ox.ac.uk/ukbiobank/).

Briefly, all imaging data was acquired on a standard Siemens Skyra 3 T scanner using the Siemens 32-channel radiofrequency receive head coil. In the T1 pipeline, tissue-type segmentation was applied using FAST (FMRIB's Automated Segmentation Tool). Global brain volumes following SIENAX analysis, subcortical and cortical volumes for 139 ROIs (Regions of Interest) were then estimated from FAST by the UK Biobank (ROIs defined in MNI152 space, based on the Harvard-Oxford cortical and subcortical atlases) (Alfaro-Almagro et al., 2018). For volumetric analyses in the current study, we investigated 3 global IDPs and, based on previous MDD and neuroinflammation literature, we identified *a priori* 2 ventricular, 6 bilateral subcortical and 10 bilateral cortical ROIs (Table 1).

**Table 1:** Regions of Interest (ROIs) for structural data.

Volumes	Thickness	Surface Area
**Cortical Bilateral**	**Cortical Bilateral**	**Cortical Bilateral**
Anterior Cingulate Gyrus	Anterior Cingulate Cortex	Anterior Cingulate Cortex
Posterior Cingulate Gyrus	Posterior Cingulate Cortex	Posterior Cingulate Cortex
Insular Cortex	Insula	Insula
Parahippocampal Gyrus	Parahippocampal Cortex	Parahippocampal Cortex
Inferior Parietal Cortex	Inferior Parietal Cortex	Inferior Parietal Cortex
Entorhinal	Entorhinal	Entorhinal
Precuneus	Precuneus	Precuneus
Lateral Prefrontal Cortex	Lateral Prefrontal Cortex	Lateral Prefrontal Cortex
Frontal Orbital Cortex	Frontal Orbital Cortex	Frontal Orbital Cortex
Frontal Medial Cortex		
**Whole Brain & Ventricles**	**Hemisphere**	**Hemisphere**
Volume of brain, grey + white matter	Global mean thickness	Global area
Volume of grey matter		
Volume of white matter		
3rd Ventricle		
4th Ventricle		
**Subcortical Bilateral**		
Hippocampus		
Ventral Striatum		
Putamen		
Pallidum		
Caudate		
Amygdala		

We also used surface area and mean cortical thickness IDPs, generated from further processing of T1 data in Freesurfer and parcellated using the Desikan-Killiany atlas. We included a hemispheric measure and cortical ROIs corresponding to the ones identified for the volumetric analysis. Smaller UK Biobank ROIs were combined for some of the ROIs used in T1 analyses (volumes, thickness, area) (see Supplement).

#### 2.4.2 Diffusion-weighted MRI (dMRI) and resting-state functional MRI (rs-fMRI)

For structural and functional connectivity, we performed whole-brain analyses, based on a recent pilot study suggesting several WM microstructural differences in psoriasis (Najafi et al., 2020).

For dMRI, we used tract measures from probabilistic tractography, performed with probtrackx/AutoPtx, following initial preprocessing (correction for eddy currents, head motion and outlier-slices) (Alfaro-Almagro et al., 2018). Mean-weighted fractional anisotropy (FA) and mean diffusivity (MD) were used as structural connectivity measures in 27 WM tracts (12 bilateral and 3 commissural).

To measure functional connection strength between brain nodes, we used the network matrices (known as “parcellated connectomes”) derived from group independent component analysis (ICA) in the pre-processed rs-fMRI data of UK Biobank participants. We analysed data from the high-dimensional (D = 100) group ICA, where each component corresponds to smaller spatial regions and can be considered as a node, as opposed to low-dimensional (D = 25) ICA, where edges represent connectivity between large networks of multiple non-contiguous spatial nodes. Cleaning of artefact nodes after visual inspection by the UK Biobank resulted in a 55x55 matrix for each participant (Cox et al., 2016). Partial correlation matrices were selected for analysis, as these give a better estimate of direct connection between nodes compared to traditional correlations (Miller et al., 2016).

### 2.5 Serum biomarkers

Biochemistry assays were collected upon recruitment. High-sensitivity serum CRP assays were analysed on a Beckman Coulter AU5800 using immunoturbidimetry (manufacturer's analytical range: 0.08–80 mg/L). There were no biochemistry data at imaging; haematology data were collected and analysed at baseline and on scan day on a Beckman Coulter.

### 2.6 Statistical analysis

#### 2.6.1 Main analysis

We analysed data using a hierarchical approach for our main, secondary and exploratory sub-analyses.

To test associations between structural ROI IDPs and psoriasis, analysis of covariance (ANCOVA) was performed using R (https://www.r-project.org/). The main predictors were psoriasis, depression and the psoriasis × depression interaction term. Other covariates included age, age^2^ (Shen et al., 2017), sex, BMI (Pearce, 2016), assessment centre, as well as head and coil position in the scanner (“table position” UK Biobank confounder set (Alfaro-Almagro et al., 2021)). Total intracranial volume was added for T1-derived ROI IDPs not normalized for head size; and handedness for bilateral ROIs. Since cortical thickness data were subject to bias depending on T1 or combined T1-T2 FLAIR input, thickness models were de-confounded for the respective data-field (26500) as recommended in (Alfaro-Almagro et al., 2021).

For the functional connectivity data, we tested associations between connectivity network matrices and psoriasis using the general linear model approach in FSLnets, with edge strength as the dependent variable, psoriasis and the psoriasis x depression term as the main independent variables of interest. We adjusted for the same covariates as for structural ROIs, as well as head motion (Alfaro-Almagro et al., 2021).

Owing to intercorrelation among diffusion metrics of different WM tracts (Cox et al., 2016), principal component analysis was used as a first step to extract latent measures as estimates of global WM integrity as well as WM structure in each of three large groups of tracts, as described in previous UK Biobank studies (Cox et al., 2016; Reus et al., 2017; Shen et al., 2017). The first unrotated principal component of FA and MD scores for each measure were entered as dependent variable in our models. Using the R prcomp function with data scaling, the proportion of variance explained by the first components was 52.6% (FA) and 47.3% (MD) for the global estimates; 51.8% (FA)/59.5% (MD), 36.8% (FA)/33.0% (MD) and 61.3% (FA)/72.3% (MD) for association and commissural fibres, projection fibres and thalamic radiations respectively.

We corrected for multiple comparisons controlling for the False Discovery Rate (FDR) (Benjamini-Hochberg method at a 0.05 threshold, using *p. adjust* in R) across all ROIs/networks within each metric (volume, thickness, area, MD, FA, connectivity edges, PCA-derived WM measures) and contrast of interest. Subsequently, for IDPs where effects of the group variable or its interaction with depression remained significant, post-hoc tests were performed using the emmeans R package. For post-hoc simple main effects, we report standardized beta coefficients and FDR-adjusted p-values.

#### 2.6.2 Psoriatic arthritis (PsA)

We further stratified patients by PsA status to explore independent effects of PsA on brain measures and re-ran the above models with a trichotomous group variable (psoriasis and PsA, psoriasis only, controls). This separate, secondary approach was chosen for PsA, because a) the subgroup of patients with joint involvement was comparatively small (n = 28) and less well-matched to the rest of the sample, and b) the PsA group may also represent the severe spectrum of skin disease in the sample, and confounding effects of psoriasis severity were not possible to disentangle. We corrected for multiple comparisons similarly to the main analyses.

#### 2.6.3 Associations with inflammatory markers

Among patients with psoriasis, we examined associations of blood biomarkers with those IDPs, where effects of either the group/psoriasis variable or its interaction with depression were significant. We used confounder-adjusted linear regression, with logarithmic transformation for CRP, and ran the models both with and without terms for depression (precuneus) and PsA (resting-state model) (for details see Supplement).

#### 2.6.4 Exploratory sub-analyses for main finding

Finally, we examined the relationship of the precuneus thickness IDP, where an interaction of psoriasis and depression was observed, with: a) the presence of lifetime passive suicidal ideation (none/once, recurrent) among depressed patients and b) psoriasis duration, using linear regression and controlling for FDR (Supplement).

#### 2.6.5 Outliers, missing data and sensitivity analyses

Models were checked for outliers via Cook's distance plots and Bonferroni outlier tests. Removing observed outliers did not significantly change the direction or size of investigated effects in any model. In two WM tracts (MD/FA in cingulate gyrus part of cingulum bilateral and middle cerebellar penducle) where a single extreme outlier was detected (value = 0, >8 median absolute deviation from median), it was attributed to measurement error and excluded from analysis as previously described (Douaud et al., 2022). Results are otherwise reported including all values. No data were missing for covariates in primary analyses except for inflammatory biomarkers, where missingness was low (∼5%) at baseline and very high (>80%) at imaging, and therefore biomarker data were not imputed. Sample sizes for sub-analyses are described in the Supplement.

As sensitivity analyses for the set of significant IDPs, we: a) adjusted additionally for alcohol use frequency, ethnicity, Townsend deprivation index, education level and hypertension history; b) investigated differences in the results among depressed participants, depending on depression phenotype. These are reported in the Supplement; results were not significantly altered (Table S7).

## 3 Results

### 3.1 Participants

The four matched groups did not differ on any of the examined sociodemographic, clinical or lifestyle characteristics, including alcohol and tobacco use at the time of scan (Table 2).

**Table 2:** Sociodemographic and clinical characteristics of participants.

Characteristics	Missing data	Psoriasis, Depressed (n = 131)	Psoriasis, Not depressed (n = 131)	Controls, Depressed (n = 393)	Controls, Not depressed (n = 393)	*p*-value^b^
**Sex, female(%)**	0 (0%)	78 (59.5%)	78 (59.5%)	234 (59.5%)	234 (59.5%)	0.999
**Age, mean(sd)**	0 (0%)	62.15 (7.33)	62.46 (7.29)	62.17 (7.36)	62.48 (7.28)	0.926
**Depression Phenotype** 0 (0%)						0.999
**CIDI**		23 (17.56%)		69 (17.56%)		
**Smith**		75 (57.25%)		225 (57.25%)		
**Both**		33 (25.19%)		99 (25.19%)		
**Handedness**						0.934
**Right**		118 (90.07%)	119 (90.84%)	351 (89.31%)	356 (90.58%)	
**Left**		10 (7.63%)	11 (8.39%)	37 (9.41%)	32 (8.14%)	
**Ambidextrous**		3 (2.29%)	1 (0.76%)	5 (1.27%)	5 (1.27%)	
**PsA presence**	0 (0%)	17 (12.9%)	11 (8.39%)			0.317
**BMI, median(IQR)**	0 (0%)	25.39 (5.77)	25.38 (5.25)	25.39 (5.92)	25.38 (5.37)	0.131
**Total GM Volume**^**a**^**, cm**^**3**^**, mean (sd)**	0 (0%)	807.42 (445.43)	796.45 (456.59)	800.79 (449.95)	801.57 (441.60)	0.256
**Total WM Volume**^**a**^**, cm**^**3**^**, mean (sd)**	0 (0%)	704.45 (384.71)	700.66 (396.86)	701.89 (394.73)	700.81 (400.97)	0.818
**Baseline Serum**						
**CRP, mg/L, median (IQR)**	53 (5.05%)	1.44 (1.69)	1.28 (2.04)	1.04 (2.01)	0.87 (1.35)	**<0.001**
**Neutrophil count, 10ˆ9 cells/L, mean (sd)**	42 (4.01%)	3.92 (1.15)	4.12 (1.24)	4.11 (1.36)	3.84 (1.20)	**0.017**
**Neutrophil count at scan, mean (sd)**	913 (87.12**%**)	4.41 (1.13)	4.81 (1.35)	4.37 (1.61)	4.00 (1.29)	0.189
**Imaging Centre**	0 (0%)					0.105
**Cheadle**		85 (64.89%)	82 (62.59%)	228 (58.01%)	228 (58.01%)	
**Reading**		8 (6.11%)	12 (9.16%)	59 (15.01%)	61 (15.52%)	
**Newcastle**		38 (29.01%)	37 (28.24%)	106 (26.97%)	104 (26.46%)	
**Ethnicity (white British)**	0 (0%)	122 (93.13%)	119 (90.84%)	357 (90.84%)	364 (92.62%)	0.729
**Townsend Deprivation Index, median(IQR)**	3 (0.29%)	−2.20 (3.57)	−2.69 (3.33)	−2.40 (3.94)	−2.40 (3.94)	0.169
**Education, university**	0 (0%)	59 (45.0%)	69 (52.7%)	213 (54.2%)	201 (51.1%)	0.333
**Alcohol use frequency, frequent drinker**	5 (0.48%)	61 (46.56%)	60 (45.80%)	178 (45.29%)	185 (47.07%)	0.819
**Smoking**	5 (0.48%)	5 (3.82%)	1 (0.76%)	10 (2.54%)	7 (1.78%)	0.207
**Hypertension**	0 (0%)	31 (23.66%)	35 (26.72%)	109 (27.73%)	80 (20.36%)	0.098
**PHQ-2 score at scan, mean (sd)**	39 (3.72%)	2.70 (1.12)	2.17 (0.55)	2.67 (1.11)	2.18 (0.59)	**<0.001**
**Lifetime recurrent passive suicidal ideation**	314 (29.96%)	32 (24.43%)	8 (6.11%)	109 (27.73%)	8 (2.03%)	**<0.001**^c^

Notes: ^a^ normalized for head size; ^b^ comparisons between four groups; ^c^ p = 0.532 between depressed groups. P-values<0.05 are reported in bold. IQR=Interquartile range, sd = standard deviation, PsA = Psoriatic arthritis, BMI=Body Mass Index, GM = grey matter, WM = white matter, CRP=C-reactive protein.

As expected, participants with psoriasis had higher baseline CRP than participants without psoriasis (p < 0.001), although this difference was borderline significant when we only examined depressed participants (p = 0.056). Patients with PsA had numerically higher CRP than patients with psoriasis only (median[interquartile range] 2.17 [3.31] versus 1.34 [1.74], p = 0.07) (Table S4). Neutrophil count at baseline was more elevated in non-depressed psoriasis patients than non-depressed controls (p = 0.05).

Lifetime depression was associated with significantly higher PHQ-2 scores at the time of imaging (p < 0.001). There was no difference in PHQ-2 scores at imaging (p = 0.77), antidepressant use (p > 0.99), longest ever depressive episode duration (p = 0.86) or number of lifetime depression episodes (p = 0.17) between depressed psoriasis patients and depressed controls. There were no differences in treatment for psoriasis (none, topical, systemic) between depressed and non-depressed patients (p = 0.87). Depressed patients tended to have shorter psoriasis duration than non-depressed participants (mean[standard deviation] 32.2 [15.9] versus 37.1 [16.5], p = 0.059) (Supplement Tables S4–S6).

### 3.2 Structural T1 MRI measures: volume, thickness and area

**Fig. 2:**
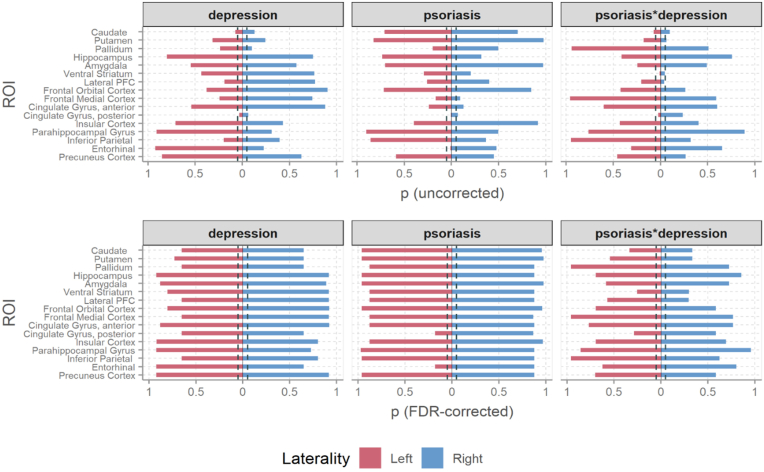
Effects on Subcortical and Cortical Volumes. Uncorrected and FDR-adjusted p for the psoriasis × depression interaction, psoriasis main effect and depression main effect. The dashed line corresponds to 0.05. PFC = prefrontal cortex.

There was no significant effect of psoriasis on any of the global, cortical or subcortical volumes. We note that, before FDR correction, the volume of the left posterior cingulate gyrus (PCG) was the only IDP where effects were significant for both the main psoriasis variable and its interaction with depression (p[uncorrected] = 0.006 and 0.023 respectively), with non-depressed psoriasis patients showing lower volumes than non-depressed controls. However, both FDR-adjusted p-values were above the 0.05 threshold (Fig. 2).

**Fig. 3:**
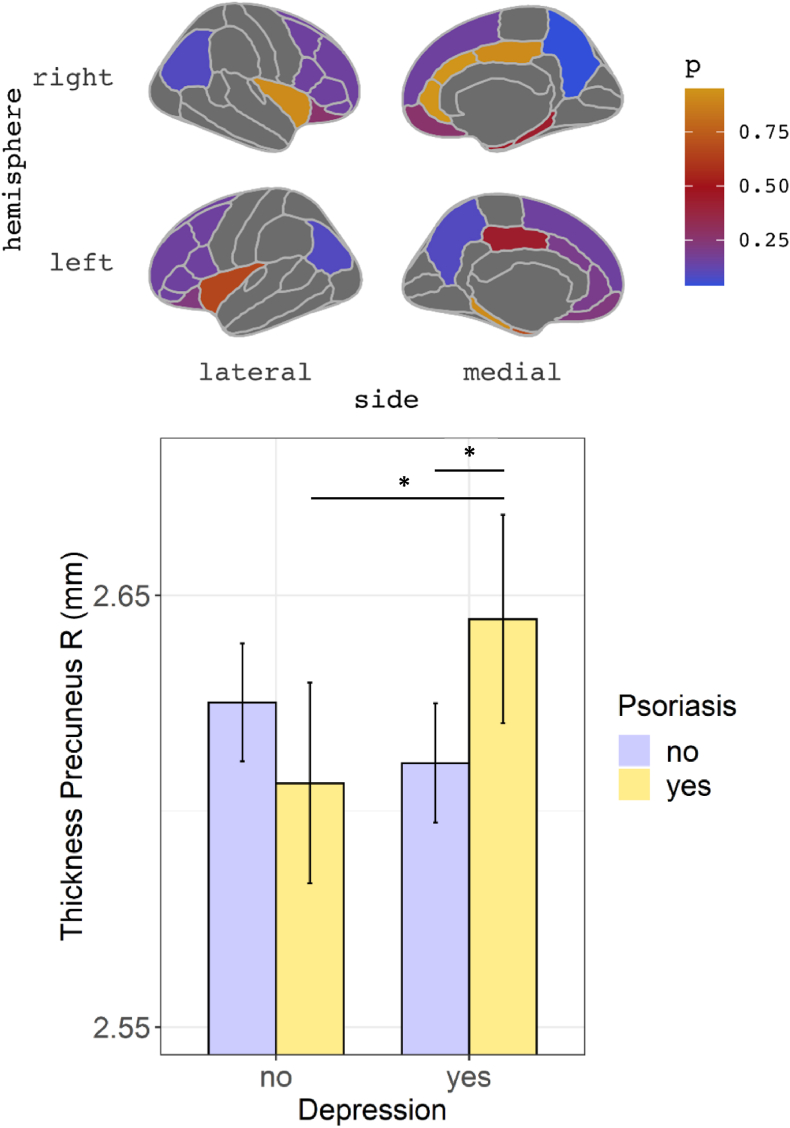
Top: FDR-adjusted p-values for interaction effects between psoriasis and depression on cortical thickness for examined ROIs; interaction was significant for thickness in the right precuneus (blue). FDR-adjusted p-values for main psoriasis or depression effects were >0.05 in all other ROIs. Parcellation according to the Desikan-Killiany atlas, used for the cortical thickness IDPs. Bottom: Barplot representing means for thickness in precuneus right in patients (yellow) and controls (lavender blue) with and without depression; errorbars represent 95% Confidence Intervals for the mean. Depressed psoriasis patients had higher precuneus thickness than both depressed controls without psoriasis and psoriasis patients without depression. Depressed patients without psoriasis had marginally lower thickness than non-depressed controls (p = 0.047). There were no effects of psoriasis among non-depressed subjects. Plot for the estimated marginal means is presented in the Supplement. (For interpretation of the references to colour in this figure legend, the reader is referred to the Web version of this article.)

We found significant psoriasis-depression interaction for the thickness of the right precuneus (*F*(1,1030) = 9.62, ω^2^ = 0.007, Cohen's *f* = 0.09; 95% confidence interval (95% CI) (0.03, 0.15), FDR-adjusted p = 0.039). Post-hoc analysis showed that patients with psoriasis had higher right precuneus thickness than patients without psoriasis, when depression was present (β = 0.26, 95% CI 0.08, 0.44; p = 0.023), but there were no differences among non-depressed participants (β = −0.15, 95% CI -0.33, 0.03; p = 0.107). Among people with psoriasis, depression was associated with higher thickness (β = 0.27, 95% CI 0.05, 0.49; p = 0.037), whereas, inversely, among people without psoriasis, depressed participants tended to exhibit lower thickness (β = −0.14, 95% CI -0.27, −0.01; p = 0.047) (Fig. 3).

Psoriasis was not associated with cortical thickness or cortical area measures in other ROIs. In further analysis, we did not find any independent effects of PsA on structural T1 measures.

### 3.3 Structural connectivity

We found no association between psoriasis and FA/MD for any of the composite or individual WM tract measures. In our secondary analysis, there was an interaction between group and depression in the global, PCA-derived MD, however post-hoc tests did not yield significant effects for psoriasis, PsA or depression. Similarly, several regions showed interactions of PsA with depression, and data visualization revealed trends of higher MD values in the left superior thalamic radiation, right cingulate gyrus part of the cingulum and right superior longitudinal fasciculus for PsA patients without depression compared to controls. However, interaction effects were not significant in any of these tracts after FDR control (see Supplement Table S9).

### 3.4 Functional connectivity

**Fig. 4:**
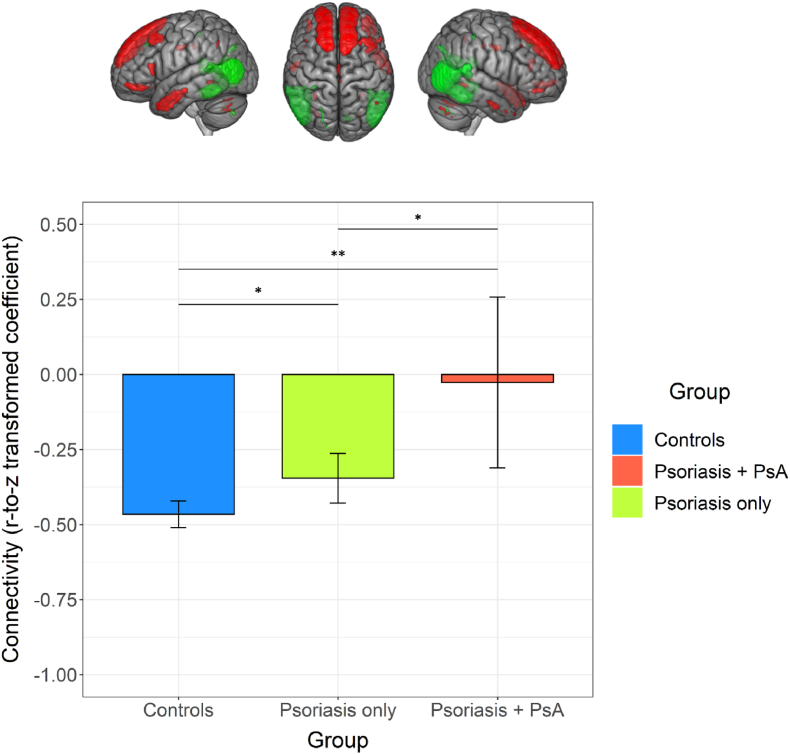
Top: Nodes 13 (red) and 41 (green) identified using group-ICA of resting-state f-MRI data of more than 4,000 UK Biobank participants (https://www.fmrib.ox.ac.uk/ukbiobank; accessed August 20, 2022). Bottom: Barplot representing means for connectivity strength measured as the z-score of the partial correlation coefficient between nodes 13 and 41; errorbars represent 95% Confidence Intervals for the mean. There were significant differences between all three groups. (For interpretation of the references to colour in this figure legend, the reader is referred to the Web version of this article.)

No differences for rs-fMRI connectivity matrices were discovered between the large group of patients with psoriasis and controls. However, when we split patients depending on joint involvement, we found significant effects of group on the edge strength between nodes 13 and 41 (Cohen's *f* = 0.13 95% CI 0.06, 0.19; FDR-adjusted p = 0.009). The group × depression interaction and depression terms were not significant. In post-hoc analysis, joint involvement was associated with decoupling between nodes 13 (mainly superior frontal area, middle temporal) and 41 (occipital and inferior temporal/temporo-occipital areas) compared to controls (β = 0.49, 95% CI 0.25, 0.74; p < 0.001) and patients with psoriasis only (β = 0.39, 95% CI 0.13, 0.64; p = 0.005) (Fig. 4). There was a smaller difference between psoriasis-only patients and controls (β = 0.11, 95% CI 0.02, 0.20; p = 0.023). The partial correlation connectome for each group is presented in Fig. S4 (Supplement).

### 3.4 Associations of brain measures with peripheral inflammation in psoriasis

Thickness in the right precuneus was not predicted by baseline CRP or neutrophil count and was not associated with neutrophil count at the time of the scan among patients with psoriasis. Similarly, there were no associations with past or present inflammatory marker levels and the connectivity strength between nodes 13 and 41 (see Supplement Table S8 and Fig. S6).

### 3.6 Associations of right precuneus thickness with suicidality and psoriasis duration

Among depressed participants with psoriasis, thickness in the right precuneus was higher in those who had recurrent passive suicidal thoughts (β = 0.43, 95% CI (0.04, 0.83), p = 0.06, before FDR control p = 0.03). Finally, we examined the effect of psoriasis duration, also given that the latter was marginally shorter for depressed versus non-depressed patients (see 3.1.); no association with right precuneus thickness was found (β = −0.004, 95% CI (−0.01, 0.002), p = 0.19).

## 4 Discussion

To the best of our knowledge, this brain imaging study includes the largest sample of psoriasis patients to date and is the first report to investigate the role of depression, PsA and systemic inflammatory burden on patients’ brain structure and connectivity.

We found higher thickness of the right precuneus cortex in comorbid patients with depression and psoriasis than in depressed physically healthy controls. Depression affected inversely psoriasis patients (thickening) and controls (thinning). In the absence of depression, psoriasis subjects tended to have thinner precuneus than controls, partly aligning with a previous study of n = 7 non-depressed psoriasis patients; in this previous sample, however, thinning also affected the parahippocampal and right cingulate apart from the superior parietal areas (Gisondi et al., 2014).

The reason for our main finding, which is the interaction between depression and psoriasis on precuneus thickness, is not clear. Cortical thickness has been proposed as a sensitive and specific marker for pathological rather than age-related brain structure changes; in the precuneus, thickness shows less individual variability than surface or volume metrics (Bruner et al., 2015; Fortea et al., 2010; Marsland et al., 2008; Szymkowicz et al., 2017). Although cortical thickness studies in depression generally find prefrontal thinning, results are less consistent for parietal regions (Schmaal et al., 2017; Suh et al., 2019). Whilst some previous work reported precuneus thickness reduction bilaterally in generally healthy older adults with late-onset MDD (Lim et al., 2012), other studies have found positive correlation between precuneus thickness and depressive symptoms in subclinical and mild-to-moderate depression, including in old (Szymkowicz et al., 2016) and young (Ducharme et al., 2014) adults, and patients with Parkinson disease (Zanigni et al., 2017).

It has been hypothesized that cortical thickness in depression may differ across disease stages, with thickening suggesting an initial compensatory production of neurotrophic factors by astrocytes following neuroinflammation (Liberto et al., 2004; Qiu et al., 2014). Whereas severe progressive neuroinflammation may ultimately result to cortical thinning, it is conceivable that reactive gliosis with maladaptive tissue remodelling may persist in some chronic inflammatory states, based on limited evidence from mild traumatic brain injury imaging studies and repetitive concussion animal models (Dall'Acqua et al., 2017), and may contribute to our results. Prior work suggests a vulnerability of precuneus and PCC to systemic inflammation; increased, CRP-associated proton density, indicating probable neuroinflammation-related oedema, was found in these regions even in the absence of MDD (Kitzbichler et al., 2021). We note a lack of association of precuneus thickening with inflammatory markers, PsA and psoriasis duration in the current study. Given the unavailability of neuroinflammation markers in our study, as well as limitations in systemic and brain inflammation measures in MDD and poor statistical correlation between the two (Enache et al., 2019), we cannot exclude a role of immunological mechanisms in our results. Nevertheless, this lack of association with inflammatory markers is at least consistent with previous PET data that did not find increases in activated microglia in MDD-free psoriasis (Hunter et al., 2016).

The differential effects of depression for precuneus may also be related to clinical phenotype differences. Szymkowicz et al. showed that right precuneus thickening in subthreshold depression is driven by somatic symptoms (Szymkowicz et al., 2017), which are pronounced in patients with chronic physical illness (Yates et al., 2004), rather than affective or cognitive depressive symptoms. In psoriasis, high levels of sleep disturbance and fatigue are found and are intertwined with psychological distress and itch (Gowda et al., 2010).

To a greater extent, our findings may reflect a synergy of depression and psoriasis in somatosensory processing among comorbid patients. The precuneus constitutes an integral part of the default mode network (DMN), participating in resting-state self-referential processing and episodic memory retrieval (Bruner et al., 2015; Szymkowicz et al., 2017). Mounting evidence suggests a key role of precuneus in itch and pain processing (Papoiu et al., 2012), including mental imagery, perception of sensory stimuli and their interaction with affective states (Mochizuki et al., 2009). Though the ACC, sensorimotor cortices and subcortical areas are primarily involved in physiological itch, itch-induced activation of the precuneus and posterior cingulate cortex has been found, which, in patients with atopic dermatitis, appears to be intense and differentiates patients’ brain itch responses from those of healthy controls (Ishiuji et al., 2009; Mochizuki et al., 2009; Papoiu et al., 2012). Depression may further modulate activity in somatosensory areas (Burkhouse et al., 2017), altering how patients with chronic inflammatory skin disease make sense of systemic symptoms such as itch, fatigue and pain. This explanation also provides a neurobiological basis for the robust correlations of depression scores with these symptoms and other subjective disease severity measures in psoriasis, which contrasts the poor correlation between depression and the objective severity of skin lesions (Lada et al., 2022).

Intriguingly, our exploratory analyses indicate a potential association of recurrent lifetime suicidality with precuneus thickening in depressed psoriasis patients. This result was marginal after FDR control, however it is noteworthy in the context of existing literature. Recent work linked cortical thickening of the right precuneus and other parietal areas to perseverative thinking, and showed that superior parietal thickness mediates the relationship between perseverative thinking and suicidality (Sheehan et al., 2022). In depression, abnormalities involving the precuneus/PCC and their connections have been associated with suicidal behaviours (Schreiner et al., 2019; Zhang et al., 2021), likely by modulating negative processing of the self, in connection to social representation deficits (Schreiner et al., 2019; Sheehan et al., 2022). Although it is unclear whether psoriasis increases suicidality risk, suicidal thinking appears to be skin-focused in over half of suicidal psoriasis patients (Dalgard et al., 2015). Furthermore, prior work suggests that suicidality, rather than depression itself, may drive neuroinflammation in some MDD patients (Holmes et al., 2018). Taking together these observations, and given that our exploratory analysis was performed in a smaller subset (n = 101), we believe that a further exploration of the role of suicidality on precuneus thickening through larger studies in psoriasis is warranted.

Using a sample of 1,048 age- and sex-matched participants, we did not find psoriasis effects on the investigated subcortical and cortical grey matter volumes, including the frontal areas and ACC, expanding results of a previous study reporting no global or hippocampal volume abnormalities in psoriasis (Pezzolo et al., 2021). Furthermore, we did not detect significant differences in WM integrity or functional connectivity in psoriasis, regardless of depression comorbidity. These latter findings partly contrast with a recent pilot report which did not find changes in global WM integrity in psoriasis, but did show higher FA among psoriasis patients in individual tracts, prominently in anterior thalamic radiations and the right cingulum (Najafi et al., 2020). One reason for this discrepancy may be the age difference between the samples. Ageing brains show robust FA decreases, with a predilection for the anterior WM (Bennett et al., 2010; Cox et al., 2016). As we investigated older adults, compared to the previous middle-aged sample, it is possible that normal WM integrity loss may have obscured subtle, psoriasis-related differences occurring in earlier life. Psoriasis severity and treatment could play a role, however this information is not available for the pilot sample. Challengingly, the previously observed FA increases suggest enhanced microstructural integrity in psoriasis; whereas, our secondary analyses showed a tendency for higher MD in the cingulum and other tracts among non-depressed patients with psoriasis and PsA, suggestive of integrity loss.

When we classified patients depending on joint involvement, we found significantly reduced resting-state connectivity between the superior frontal and temporo-occipital cortices for psoriasis patients with PsA compared to patients without PsA and, in particular, compared to controls without psoriasis. Fronto-occipital connections are implicated in visuospatial processing and motor function (Makris et al., 2005), whilst impaired connectivity of long-distance tracts, such as the fronto-occipital and longitudinal fasciculi, which link several areas involved in pain perception and attention, has been associated with chronic pain (Geha et al., 2008; Rayhan et al., 2013). Emerging evidence suggests that systemic inflammation leads to disruption of fronto-occipital WM tract integrity in humans (Ho et al., 2022; Tagliapietra and Monaco, 2020; Zheng et al., 2021). Neutrophils and CRP did not correlate with edge strength in the current study; however, our PsA sample was small and larger studies are needed to replicate this result. We did not find an independent role for depression in the fronto-temporo-occipital decoupling.

The major strengths of our study are the large sample size, the multi-centre, national cohort, and the use of age- and sex-matched controls within the UK Biobank dataset. Selection bias within the UK Biobank was mitigated by making use of the whole resource, and not restricting data to MHQ respondents or linkage (Davis et al., 2020).

Our findings need to be interpreted in the light of some limitations. Although the validity of included psoriasis and PsA samples is supported by the prevalence of cases in the cohort and the blood biomarker data, misclassification cannot be excluded. Similarly, depression definitions may not correspond to fully met MDD criteria in all cases, although every effort was made to eliminate effects of heterogeneity in depression for case-control inference. Second, UK Biobank recruits tend to have good health status and treatment data in our sample suggest overall mild psoriasis severity, so that our findings may not be generalizable in severe disease. Third, temporal and thereby causal relations between MDD, psoriasis and brain IDPs cannot be ascertained within the present dataset. We note that the study was designed to elucidate effects of psoriasis and depression on the brain, independent of significant cardiometabolic morbidity; cardiometabolic disease shows genetic overlap with and predisposition to psoriasis (Wu et al., 2022) and is expected to confound the investigated associations. However, we cannot exclude further synergistic effects among psoriasis, depression and cardiometabolic disorders in the context of chronic inflammatory states, which are beyond the scope of the current study. Finally, CRP and neutrophil counts may vary considerably over time and not adequately reflect patients’ overall inflammatory load. There is currently no gold standard for markers of systemic inflammation in psoriasis. We report blood biomarkers at a second time-point in a small subset, as well as exploratory results for PsA, which can be considered a proxy for a higher chronic inflammatory state; however, there was high missingness for markers at the time of scan and serum levels of proinflammatory cytokines involved in psoriasis pathogenesis, such as IL-17, are not measured in the UK Biobank.

## 5 Conclusions

In conclusion, this study provides new evidence on the neuroanatomical underpinnings of the link between psoriasis and depression. We found precuneus thickening in depressed patients with psoriasis, which may reflect comorbidity-specific somatosensory processing and cognitive processes related to lifetime suicidal thinking. No effects of psoriasis on subcortical or cortical volumes, surface area and WM integrity were found. PsA may alter resting-state connectivity in psoriasis. We did not find evidence to support a direct role of systemic inflammation in psoriasis and PsA effects on the brain; though a role for neuroinflammation in depressed psoriasis patients cannot be excluded. Comorbid depression in psoriasis is undetected in over half of cases (Dalgard et al., 2018); understanding the neurobiological mechanisms underlying mood in these complex patients is important to ensure early intervention and tailored therapies, ultimately improving patients’ quality of life. Future research is needed to confirm the replicability of these findings in severe disease and larger PsA populations, and further explore the role of suicidality and neuroinflammation, for example using markers of microglia activation.

## Role of the funding source

The funding body had no involvement in study design, writing of the report, or data collection, analysis, or interpretation.

## Declaration of competing interest

The authors declare the following financial interests/personal relationships which may be considered as potential competing interests: G Lada reports a relationship with Janssen that includes: speaking and lecture fees. G Lada reports a relationship with Lilly that includes: speaking and lecture fees. G Lada reports a relationship with Leo that includes: speaking and lecture fees. G Lada reports a relationship with Novartis that includes: speaking and lecture fees. CE Kleyn reports a relationship with Janssen that includes: funding grants and speaking and lecture fees. CE Kleyn reports a relationship with Eli Lilly that includes: funding grants and speaking and lecture fees. CE Kleyn reports a relationship with 10.13039/100004336Novartis that includes: funding grants and speaking and lecture fees. CE Kleyn reports a relationship with 10.13039/100004319Pfizer that includes: consulting or advisory, funding grants, and speaking and lecture fees. CE Kleyn reports a relationship with L'oreal that includes: consulting or advisory, funding grants, and speaking and lecture fees. CE Kleyn reports a relationship with Almirall that includes: speaking and lecture fees. CE Kleyn reports a relationship with Leo that includes: speaking and lecture fees. CE Kleyn reports a relationship with 10.13039/100006483Abbvie that includes: speaking and lecture fees. CE Kleyn reports a relationship with 10.13039/100011110UCB that includes: speaking and lecture fees. H Chinoy reports a relationship with 10.13039/100004336Novartis that includes: board membership, consulting or advisory, funding grants, and speaking and lecture fees. H Chinoy reports a relationship with 10.13039/100011110UCB that includes: board membership, consulting or advisory, funding grants, and speaking and lecture fees. H Chinoy reports a relationship with 10.13039/501100004628MedImmune that includes: funding grants. H Chinoy reports a relationship with Orphazyme that includes: board membership, consulting or advisory, and speaking and lecture fees. H Chinoy reports a relationship with Lilly that includes: board membership, consulting or advisory, and speaking and lecture fees. H Chinoy reports a relationship with 10.13039/100005614Biogen that includes: board membership, consulting or advisory, and speaking and lecture fees. H Chinoy reports a relationship with Abbvie that includes: travel reimbursement. H Chinoy reports a relationship with Janssen that includes: travel reimbursement. RB Warren reports a relationship with 10.13039/100006483AbbVie that includes: consulting or advisory and funding grants. RB Warren reports a relationship with Almirall that includes: consulting or advisory and funding grants. RB Warren reports a relationship with 10.13039/100002429Amgen that includes: consulting or advisory and funding grants. RB Warren reports a relationship with 10.13039/100006436Celgene that includes: consulting or advisory and funding grants. RB Warren reports a relationship with Janssen that includes: consulting or advisory and funding grants. RB Warren reports a relationship with Lilly that includes: consulting or advisory and funding grants. RB Warren reports a relationship with Leo that includes: consulting or advisory and funding grants. RB Warren reports a relationship with Biogen that includes: consulting or advisory. RB Warren reports a relationship with 10.13039/100004336Novartis that includes: consulting or advisory and funding grants. RB Warren reports a relationship with 10.13039/100004319Pfizer that includes: consulting or advisory and funding grants. RB Warren reports a relationship with 10.13039/100011110UCB that includes: consulting or advisory and funding grants. RB Warren reports a relationship with Arena that includes: consulting or advisory. RB Warren reports a relationship with Astellas that includes: consulting or advisory. RB Warren reports a relationship with Avillion that includes: consulting or advisory. RB Warren reports a relationship with Boehringer Ingelheim that includes: consulting or advisory. RB Warren reports a relationship with Bristol Myers Squibb that includes: consulting or advisory. RB Warren reports a relationship with DiCE that includes: consulting or advisory. RB Warren reports a relationship with GSK that includes: consulting or advisory. RB Warren reports a relationship with Sanofi that includes: consulting or advisory. RB Warren reports a relationship with Sun Pharma that includes: consulting or advisory. RB Warren reports a relationship with Union that includes: consulting or advisory.

## Data Availability

The authors do not have permission to share data.
