# Supplementary material for: Brain structure and connectivity in psoriasis and associations with depression and inflammation; findings from the UK biobank
Source: Brain Behav Immun Health. 2022 Nov 21;26:100565. doi: 10.1016/j.bbih.2022.100565 (PMC9719019; doi:10.1016/j.bbih.2022.100565)
Supplement: Multimedia component 1 [file mmc1.docx]

**Supplementary Material**

Table of Contents

[A. Participants 2](#_Toc105064910)

[1. General Exclusion Criteria 2](#_Toc105064911)

[2. Definitions 4](#_Toc105064912)

[2.1. Defining Participants with Psoriasis and Non-psoriasis Controls 4](#_Toc105064913)

[2.2. Defining Participants with Depression and Non-depressed Controls 8](#_Toc105064914)

[3. Diffusion-weighted and resting-state fMRI samples 10](#_Toc105064915)

[B. Magnetic Resonance Imaging (MRI) 11](#_Toc105064916)

[1. T1-weighted MRI 11](#_Toc105064917)

[2. Diffusion-weighted MRI 12](#_Toc105064918)

[3. Resting-state functional MRI (rs-fMRI) 14](#_Toc105064919)

[C. Statistical analysis - Inflammatory markers and Sub-analyses 14](#_Toc105064920)

[D. Results - Baseline Characteristics 15](#_Toc105064921)

[1. Clinical characteristics 15](#_Toc105064922)

[2. Psoriatic Arthritis subgroup 16](#_Toc105064923)

[2. Diffusion and resting-state fMRI samples 16](#_Toc105064924)

[E. Results - The rs-fMRI connectome 17](#_Toc105064925)

[F. Results - Sensitivity Analyses 18](#_Toc105064926)

[G. Additional Figures and Tables 19](#_Toc105064927)

## A. Participants

### 1. General Exclusion Criteria

Participants having any of the self-reported diagnoses in Criterion A., any of the inpatient diagnoses in Criterion B., meeting Criterion C. or D. were excluded from analysis.

| **Criterion A. Self-reported diagnoses by time of scan in Data-Fields 20001, 20002** | |
| --- | --- |
| Cancer | any cancer (any code in Field 20001) |
| Cardiovascular | angina, heart attack/myocardial infarction, heart failure/pulmonary oedema, peripheral vascular disease, stroke, ischaemic stroke, transient ischaemic attack (TIA), subdural haemorrhage/haematoma, subarachnoid haemorrhage |
| Pulmonary | respiratory failure |
| Gastro/ Hepatobilary | pancreatitis, inflammatory bowel disease, Crohn’s disease, ulcerative colitis, oesophageal varices, hepatitis, non-infective hepatitis, liver failure/cirrhosis, |
| Renal | renal/kidney failure, renal failure requiring dialysis, renal failure not requiring dialysis |
| Endocrinological | diabetes, type 1 diabetes, type 2 diabetes, parathyroid gland problem (not cancer), parathyroid hyperplasia/adenoma, disorder of adrenal gland, adrenal tumour, adrenocortical insufficiency/Addison’s disease, phaeochromocytoma, disorder or pituitary gland, pituitary adenoma/tumour, Conn’s, Cushing’s, thyroiditis, Grave's disease |
| Neurological | neurological injury/trauma, infection of nervous system, brain abscess/intracranial abscess, encephalitis, meningitis, spinal abscess, cranial nerve problem/palsy, Bell's palsy/facial nerve palsy, spinal cord disorder, paraplegia, peripheral nerve disorder, peripheral neuropathy, acute infective polyneuritis/Guillain-Barre syndrome, chronic/degenerative neurological problem, motor neurone disease, myasthenia gravis, multiple sclerosis, Parkinson’s disease, dementia/Alzheimer’s/cognitive impairment, epilepsy, migraine, head injury, spinal injury, cerebral palsy, optic neuritis |
| Psychiatric | schizophrenia, mania/bipolar disorder/manic depression, alcohol dependency, opioid dependency, other substance abuse/dependency (not incl. nicotine), post-traumatic stress disorder, obsessive compulsive disorder |
| Musculoskeletal | connective tissue disorder, rheumatoid arthritis, systemic lupus erythematosis/SLE, ankylosing spondylitis, bursitis, sunovitis, osteomyelitis, myositis/myopathy, sarcoidosis, vasculitis, giant cell/temporal arteritis, polymyalgia rheumatica, Wegener’s granulmatosis, polyartertis nodosa, Sjogren's syndrome/sicca syndrome, dermatopolymyositis, dermatomyositis, polymyositis, scleroderma/systemic sclerosis, microscopic polyarteritis |
| Dermatological | pemphigoid/pemphigus, eczema/dermatitis, lichen planus, lichen sclerosis, urticaria |
| Infections | HIV/AIDS, infective/viral hepatitis, hepatitis b, hepatitis c |

| **Criterion B. ICD-9 and ICD-10 codes in a hospital admission record (Data-Fields** **41270, 41271)** | |
| --- | --- |
| Psychiatric | Dementia and any mental disorders due to known physiological conditions (delirium etc.), any substance dependence (apart from nicotine), schizophrenia/psychotic disorder, bipolar/schizoaffective disorder/mania/cyclothymia, intellectual disabilities (ICD-10: F0, F11.2, F12.2, F13.2, F14.2, F16.2, F2, F30, F31, F34.0, F7; ICD-9: 290, 295, 296, 2911, 2912, 2941, 303, 304) |
| Neuro/ Cerebrovascular | All vascular syndromes of brain in cerebrovascular diseases, nontraumatic subarachnoid haemorrhage, nontraumatic intracerebral haemorrhage, other and unspecified nontraumatic intracranial haemorrhage, cerebral infarction, other cerebrovascular diseases, Parkinson’s, dementia, multiple sclerosis (ICD-10: G45, G46, G30, G31, G35, G20, I60, I61, I62, I63, I64, I67; ICD-9: 332, 340, 3409, 43) |
| Diabetes Mellitus (ICD-10: E10, E11) | |
| Inflammatory Bowel Disease (IBD) (ICD-10: K50, K51) | |
| Atopic dermatitis (ICD-10: L2) | |
| Other and unspecified dermatitis (ICD-10: L3) | |

| **Criterion C. “Probable Bipolar I or II” according to Smith et al. (Smith et al., 2013) or any mania, bipolar or schizoaffective diagnosis in any source (Data-Fields** **130885; 130891;** **130893)** |
| --- |
|  |
| **Criterion D. Self-reported having ever been diagnosed with psychotic disorder/schizophrenia or manic depression in online follow-up mental health questionnaire-MHQ (by any professional)** |

Note on general exclusion criteria

Criterion A. is the main exclusion criterion (data completeness); this information was given by all participants over three separate time points by the time of the scan. Non-cancer illness names correspond to the UK Biobank data-coding 6, which can be found online for the whole cohort (https://biobank.ndph.ox.ac.uk/showcase/field.cgi?id=20002; accessed 20 August 2022).

Criterion B. adds a second filter for selected self-reported comorbidities; these are likely to have the greatest effects on brain outcome measures; or increase misclassification for depression/psoriasis; or are classic comorbidities of psoriasis (diabetes, IBD), and may confound its inflammatory effects on the brain.

Criterions C. and D. add an additional filter to exclude patients with bipolar disorder and schizophrenia.

### 2. Definitions

### 2.1. Defining Participants with Psoriasis and Non-psoriasis Controls

Diagnostic Sources

We used all three main sources of diagnostic data in the UK Biobank for psoriasis and psoriatic arthritis (PsA):

- **Self-reported diagnoses**, collected at three instances (baseline, online follow-up and at scan). Participants reported having psoriasis/PsA in an interview with the researcher, after being asked the following question: “In the touch screen [a previously completed questionnaire] you selected that you have been told by a doctor that you have other serious illnesses or disabilities, could you now tell me what they are?” (https://biobank.ndph.ox.ac.uk/showcase/refer.cgi?id=100235; accessed 20 August 2022)
- **Hospital inpatient admissions**, obtained through linkage to external data providers. Diagnoses were coded in either ICD (International classification of diseases)-10 or, for earlier dates, ICD-9.
- **Primary care data**, available through linkage for approximately 50% of the cohort at the time of our data cut-off.

Psoriasis Definition

Given the higher potential for misclassifying psoriasis based on self-report (e.g. versus other inflammatory skin condition) rather than a physician diagnosis, as well as the limited availability of data from other sources (primary care data for only half of the cohort, hospital data from inpatient admissions only), the inclusion criteria for our psoriasis sample were:

1. Psoriasis in primary care (GP) or hospital records coded by the time of the MRI scan,

**OR**

1. Self-reported diagnosis of psoriasis by the time of scan, if **at least one** of the following criteria was also met:
   1. psoriasis in hospital or primary care records at any date or missing date data
   2. a diagnosis of PsA in any source
   3. self-reported treatment for psoriasis by the time of the MRI scan (Fig. S1)

PsA Definition

Due to the low prevalence of PsA, we used a broader criterion to define PsA in our secondary analyses, including participants with a PsA diagnosis in any of the three sources by the time of the scan (self-report, hospital and primary care).


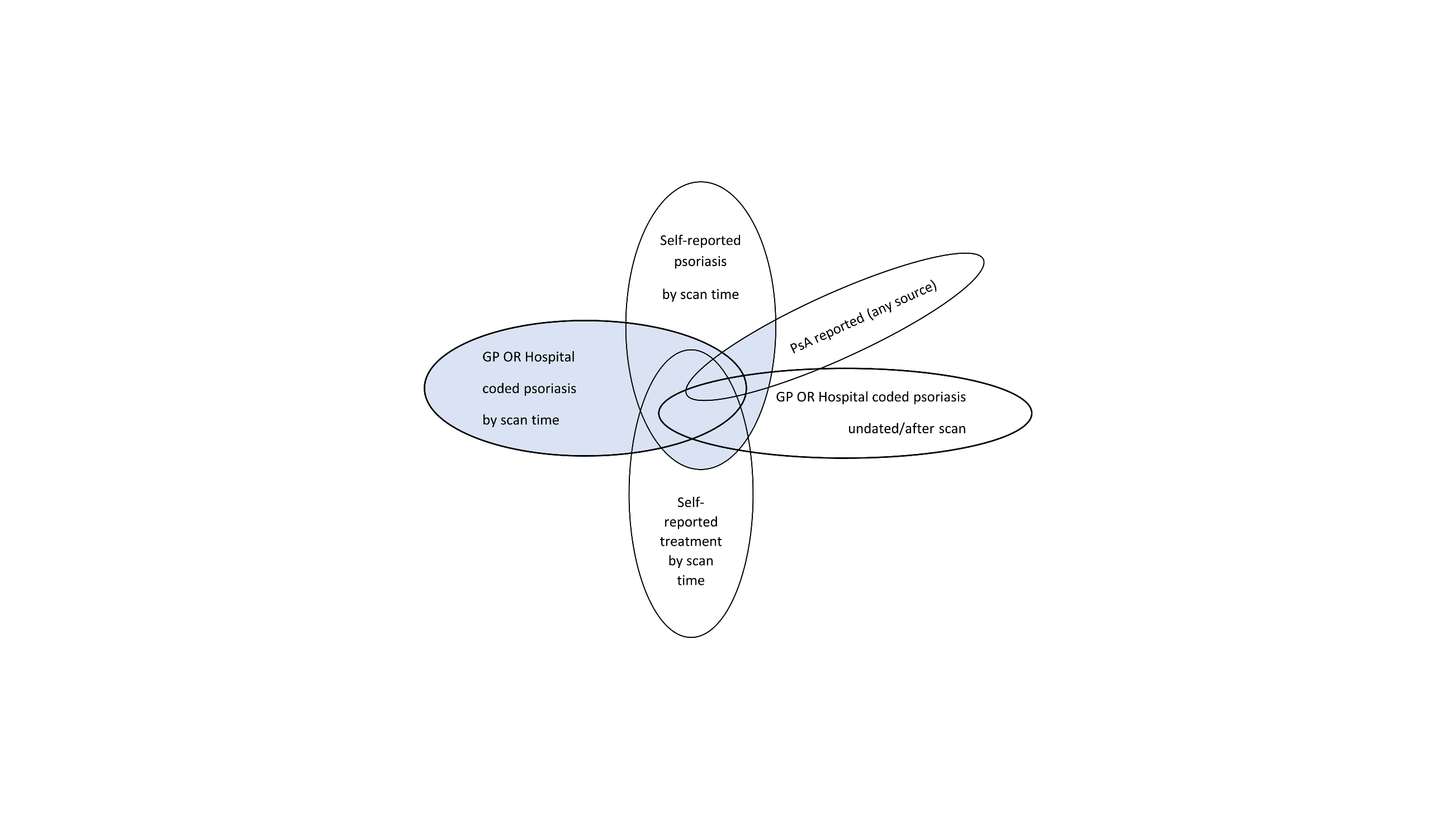


**Figure S1:** Venn diagram representing the criteria used for defining psoriasis. The light blue area represents participants meeting the criteria for the psoriasis definition. The sizes of the shapes and overlapping regions are arbitrary.

ICD-codes

Filtering for primary care and hospital records in the present study was based on ICD-9 and ICD-10 codes for psoriasis and, for our secondary analysis, PsA (Table S1). Because a minority of PsA cases present without psoriasis, participants with codes for PsA but no evidence for skin psoriasis were excluded.

|  | ICD-10 | ICD-9 |
| --- | --- | --- |
| Psoriasis | L40.0 Psoriasis vulgaris | 696.1 Other psoriasis |
|  | L40.1 Generalised pustular psoriasis |  |
|  | L40.3 Pustulosis palmaris et plantaris |  |
|  | L40.4 Guttate psoriasis |  |
|  | L40.8 Other psoriasis |  |
|  | L40.9 Psoriasis, unspecified |  |
| Psoriatic arthritis | L40.5 Arthropathic psoriasis  M07.0 Distal interphalangeal psoriatic arthropathy  M07.2 Psoriatic spondylitis (Multiple sites)  M07.3 Other psoriatic arthropathies | 696.0 Psoriatic arthropathy |
| Note: There was no record in the database corresponding to an L40.2 diagnosis (Acrodermatitis continua). | | |

**Table S1: ICD (International Classification of Diseases) codes used**

Self-reported treatment

For the psoriasis/control criteria and sub-analysis, we included UK Biobank codes for current treatment reported by participants at any UK Biobank visit up until (and including) the day of the scan, corresponding to topical coal tar products, dithranol, salicylic acid, topical calcineurin inhibitors, vitamin D analogues, systemic treatments (acitretin, methotrexate, ciclosporin) and biologics (adalimumab and efalizumab). We also included topical steroid codes, in line with previous psoriasis research in the UK Biobank (Glanville et al., 2021b) and because the likelihood for these to be used for indications other than psoriasis in patients was considered very low, given our psoriasis definition and general exclusion criteria (described in Supplement A.1).

The codes included (UK Biobank Data-Field 20003) are as follows:

| 1140879902, 1140879904, 1140879906, 1140879910, 1140879922, 1140879926, 1140879928, 1140879930, 1140879934, 1140879938, 1140879940, 1140879942, 1140880000, 1140880002, 1140880004, 1140878412, 1140878414, 1140878416, 1140878422, 1140878428, 1140878430, 1140878432, 1140878434, 1140878436, 1140878438, 1140878440, 1140878442, 1140878444, 1140878446, 1140878448, 1140878450, 1140878452, 1140878454, 1140878456, 1140878458, 1140878508, 1140883740, 1140916276, 1140878408, 1141167140, 1140878426, 1140878498, 1140878504, 1140878378, 1140878350, 1140878346, 1140878348, 1140880092, 1140880102, 1140911642, 1141179842, 1141186960, 1140923732, 1140923728, 1141180200, 1140883820, 1140923732, 1141187336, 1141187338, 1141179982, 1141179992, 1140879942, 1140880090, 1141174388, 1140880086, 1141152236, 1141188588, 1141188594, 1140869848, 1141181020, 1140910036, 1141167744, 1140870016, 1140869870, 1140910382, 2018943436, 2018943438, 1140869952, 2038459888, 2038459890, 1140879916, 1140879918, 1140878420, 1140878494, 1140878486, 1140882740, 1140882852, 1140882732, 1140882898, 1140882742, 1140888630, 1140882822, 1140884642, 1140884646, 1140882150, 1140921024, 1140882694, 1140882630, 1140882618, 1140926988, 1140909894, 1140854700, 1140909894, 1140884716, 1140884636, 1140884640, 1140854834, 1140854816, 1140854990, 1140909952, 1140854916, 1140888172, 1140888176, 1140888178, 1140888184, 1140864820, 1140909786, 1140874930, 1140884654, 1140870954, 1140874790, 1140882800, 1140888092, 1140882910, 1140884700, 1140868364, 1141157402, 1140913292, 1140878512, 1140888074, 1140882794, 1140910884, 1140864184, 1141173346, 1141201130, 1140878226, 1140878418, 1140882782, 1140884700, 1140884704, 1140888098, 1141145710, 1141167140, 1141179992, 1140880018, 1140880058, 1140880060, 1140880270, 1140880272, 1140882622, 1140916272, 1140916274, 1141164754, 1140888130, 1140888628 |
| --- |

Primary Care Data Mapping

For the primary care dataset, diagnostic data were mapped to ICD-codes from read codes (versions 2 and 3). To minimize misclassification, we performed the mapping using the linked primary care dataset as follows: the individual ICD and Read code browsers, and the clinical code mapping lists of the UK Biobank (produced using TRUD-Technology Reference Update Distribution and NHSBSA-NHS Business Services Authority resources, see <https://biobank.ndph.ox.ac.uk/showcase/showcase/docs/primary_care_data.pdf>; accessed 20 August 2022) were manually inspected, the mapping status and flags for each code annotated by the UK Biobank was evaluated, and all the appearances of each code within the diagnostic and mapping lists were cross-examined, where more than one mappings were possible.

Only read codes mapping exclusively to ICD codes for psoriasis (L40 codes) were included. We excluded participants with entries mapping exclusively to PsA but not psoriasis (L405, M070A, M072A, M073A or 696.0). For codes with discrepancies between ICD-9 and ICD-10 mapping, only relevant entries were included (e.g. read 3 X506Y entries dated before 1995 (ICD-9) were excluded). To avoid introducing bias by further data cleaning, quality checks were performed after this initial filtering and before matching: no participants with codes for psoriasis in the sample had likely erroneous dates of diagnoses identified by the UK Biobank (code: 01/01/1901; 02/02/1902; 03/03/1903; 07/07/2037). Given the retirement of read codes (last release of CTV3 in 2018), we confirmed that no participant had a diagnosis date after 2017. Furthermore, all participants included in the sample were also identified by the UK Biobank to have at least one code corresponding to psoriasis “L40-code” across the three diagnostic sources (Data-Field 131743). A simple list of Read 2 and Read 3 to ICD mapping by the UK Biobank can be found under data-codings 1834 and 1835 (<https://biobank.ndph.ox.ac.uk/ukb/coding.cgi?id=1834&nl=1>;<https://biobank.ndph.ox.ac.uk/ukb/coding.cgi?id=1835&nl=1>). In our matched dataset, the following psoriasis codes are encountered among cases with primary care records (regardless of whether they also have self-reported/ hospital-coded psoriasis): read 2: M161z, M1610, M161., M16y0; read 3: M161z, X506i, X506Y, M1616, M1610, Myu30, M1614, X506b, X506m, XaYOx, XaNXt, M1611, X506Z, M161D, M161., XaYOv.

We performed a similar process to map read codes to ICD codes for PsA. Out of all codes mapping to PsA, the following were identified in our matched psoriasis sample: M160., M160z.

Diagnostic sources in matched sample

We summarize the main diagnostic sources for participants with psoriasis and PsA in the total matched sample below. As expected, inpatient reports were higher among those with comorbid PsA (Table S2):

**Table S2: Percentage of participants in the total matched sample having a diagnosis of psoriasis and PsA in each of the three main diagnostic sources**

| Diagnostic Source | Psoriasis (n=262) | PsA (n=28) |
| --- | --- | --- |
| Primary care | n=177 (67.5%) | n=17 (60.7%) |
| Hospital admissions | n=38 (14.5%) | n=6 (21.4%) |
| Self-report | n=160 (61.0 %) | n=17 (60.7%) |

Controls for psoriasis

As controls, we used participants who had never had a diagnosis of psoriasis or PsA in the available primary care or hospital admission records or self-reported psoriasis or PsA or used treatments indicated for psoriasis by the time of scan. None of these participants reported or received a diagnosis for psoriasis at an unknown date or after the scan, by the time of our data cut-off. In order to define controls in the primary care records, we excluded any possible or probable psoriasis or PsA entries, including read 2 and read 3 codes with mappings not exclusive for psoriasis or PsA (e.g. parent codes corresponding to higher hierarchical levels in ICD taxonomy or codes matching to both one psoriasis ICD code and one or more ICD codes for other illnesses).

### 2.2. Defining Participants with Depression and Non-depressed Controls

Depression Definition

Included are those participants who meet, by the time of MRI scan, criteria for:

1. the “CIDI” UK-Biobank phenotype (Davis et al., 2020) OR
2. the “probable major depressive disorder (MDD)- Smith” phenotype (Glanville et al., 2021a; Smith et al., 2013)

**a. CIDI Phenotype**

*In the online follow-up questionnaire dated any time before or on the day of the MRI scan, participant reported having ever had*:

Sadness/Depression for ≥ 2 weeks (Data-Field 20446 = 1)

**OR**

Loss of interest for ≥ 2 weeks (Data-Field 20441 = 1)

**AND** For most of day or all day long (Data-Field 20436 ≥ 3)

**AND** Almost every day or every day (Data-Field 20439 ≥ 2)

**AND** At least somewhat impairment (Data-Field 20440 ≥ 2)

**AND** Total number of symptoms endorsed (incl. core) ≥ 5:

- Sadness/Depression (core) (Data-Field 20446 = 1);
- Loss of interest (core) (Data-Field 20441 = 1);
- Tired / low energy (Data-Field 20449 = 1);
- Gain / loss of weight (Data-Field 20536 ≥ 1);
- Sleep change (Data-Field 20532 = 1);
- Trouble concentrating (Data-Field 20435 = 1);
- Feeling worthless (Data-Field 20450 = 1);
- Thinking about death (Data-Field 20437 = 1)

**b. Probable major depressive disorder (MDD) “Smith”:**

*At baseline, at follow-up or at time of scan has self-reported:*

(i) Ever been depressed for a whole week (Data-Field 4598 = 1) (mood core)

**AND** Duration ≥ 2 weeks (Data-Field 4609 ≥ 2) AND

**AND** ≥ 1 episode (Data-Field 4620 ≥ 1) AND

**AND** ever seen a GP OR a psychiatrist for nerves, anxiety, depression (Data-Fields 2090 = 1 OR 2100 = 1)

**OR**

(ii) Ever been unenthusiastic/disinterested for a whole week (Data-Field 4631 = 1) (anhedonia core)

**AND** Duration ≥ 2 weeks (Data-Field 5375 ≥ 2)

**AND** ≥ 1 episode (Data-Field 5386 ≥ 1)

**AND** ever seen a GP *OR* a psychiatrist for nerves, anxiety, depression (Data-Fields 2090 = 1 OR 2100 = 1)

Non-depressed controls

UK Biobank participants who meet the following criteria (by the time of scan) were included as non-depressed controls:

| **NOT** |  | “CIDI” UK-Biobank phenotype |
| --- | --- | --- |
| **AND NOT** |  | probable major depressive disorder (MDD) “Smith” phenotype |
| **AND NOT** |  | self-reported depression in main questionnaire (1286 code in Data-Field 20002; any instance up to scan) |
| **AND NOT** |  | self-reported depression in mental health questionnaire (MHQ) online follow-up (33407 code in Data-Field 20544) |
| **AND NOT** |  | code for depression (ICD F32x, F33x) in hospital admission records |
| **AND NOT** |  | code for depression in primary care records (read 2 and read 3 codes mapped to ICD F32x, F33x) |
| **AND NOT** |  | ever sought help for nerves/anxiety/depression by a GP/ psychiatrist (Data-Fields 2090, 2100) |
| **AND NOT** |  | ever attempted suicide (Data-Field 20483) |
| **AND NOT** |  | self-reported anxiety/panic attack OR nervous breakdown OR chronic fatigue (codes 1482, 1286, 1287 and 1288 in Data-Field 20002) |
| **AND NOT** |  | self-reported ever taking antidepressants or antipsychotics |
| **AND NOT** |  | have PHQ-9 score > 5 (as part of the MHQ, derived from Data-Fields: 20514, 20510, 20517, 20519, 20511, 20507, 20508, 20518, 20513) |
| **AND NOT** |  | have had >1 or unknown number of episodes of isolated low mood or isolated disinterest or prolonged episodes that continue into one another (to exclude dysthymia/subclinical disorders) (Data-Field 20442, MHQ) |

Primary Care mapping for non-depressed control criteria

We conducted mapping from read 2 and read 3 codes to ICD-10 depression codes using an approach similar to the one described for psoriasis controls. To define non-depressed controls, we excluded all participants with any of the following codes in primary care:

Read 2: Eu32., Eu320, Eu321, Eu322, Eu323, Eu324, Eu325, Eu326, Eu327, Eu328, Eu329, Eu32A, Eu32B, Eu32y, Eu32z, Eu33., Eu330, Eu331, Eu332, Eu333, Eu334, Eu33y, Eu33z

Read 3: 2257., E0043, E1121, E1122, E1123, E1124, E1126, E1131, E1132, E1133, E1134, E1135, E1136, E1137, E1137, E11y2, E130., E2B0., E2B1., Eu320, Eu321, Eu322, Eu323, Eu32y, Eu32z, Eu330, Eu331, Eu332, Eu333, Eu334, Eu33y, Eu33z, X00SO , X00SQ, X00SR, X00SS, X00SU, X00Qy, XE1Y0, XE1Y1, XE1YC, XE1YC, XE1ZY, XE1ZZ, XE1Za, XE1Zb, XE1Zc, XE1Zc, XE1Zd, XE1Ze, XE1Zf, XSEGJ, XSGok, XSGol, XSGom, XSGon, XaB9J, XaCHo, XaCHr, XaCHs, XaCIs, XaCIt, XaCIu, XaX53, XaX54, XaY2C, XM1GC, Xa0wV

Antidepressant and antipsychotic treatment (non-depressed control criteria)

Regarding antidepressants, we used the fifty two treatment codes identified by Glanville *et al.* (Glanville et al., 2021a) and expanded those, additionally including (Data Field 20003):

| 1140879624, 1140879632, 1140879552, 1140916288, 1140867916, 1140867806, 1140867824, 1140867712, 1140867720, 1140867722, 1140867734 |
| --- |

For antipsychotics, we identified codes similar to Glanville *et al.* (Glanville et al., 2021a).

Depression Phenotype as Matching Variable

During matching, we matched depressed psoriasis patients to depressed non-psoriasis controls for depression phenotype, using three categories: “Smith only”, “CIDI only” and, for those who met both definitions, “both”. This approach was adopted to account for possible selection bias of completing the mental health questionnaire (CIDI) (Davis et al., 2020).

### 3. Diffusion-weighted and resting-state fMRI samples

Some participants of the T1 MRI dataset were missing dMRI and rs-fMRI data and samples for each sequence are presented in Table S3.

| **Table S3: Samples for MRI sequences** | | | | |
| --- | --- | --- | --- | --- |
| MRI datasets | Ps-Dp | Ps-NDp | Dp-NPs | NPs-NDp |
| T1 MRI | 131 (17 PsA) | 131 (11 PsA) | 393 | 393 |
| Diffusion MRI | 127 (16 PsA) | 126 (11 PsA) | 371 | 375 |
| Resting-state fMRI | 127 (16 PsA) | 127 (11 PsA) | 373 | 380 |
| Notes: Ps=psoriasis, Dp=depression, NPs=no psoriasis, NDp=no depression, PsA=psoriatic arthritis | | | | |

## B. Magnetic Resonance Imaging (MRI)

### 1. T1-weighted MRI

Image acquisition and pre-processing performed by the UK Biobank is presented here in more detail. All imaging data was acquired on a standard Siemens Skyra 3T scanner using the Siemens 32-channel radiofrequency receive head coil and were processed using FSL software (Alfaro-Almagro et al., 2018). T1 MRI images were initially defaced. Following gradient distortion correction (GDC) using BET (Brain Extraction Tool) (Jenkinson et al., 2005) and FLIRT (FMRIB's Linear Image Registration Tool), images were non-linearly warped to MNI152 space (using FMRIB's Nonlinear Image Registration Tool FNIRT). Tissue-type segmentation was applied using FAST (FMRIB's Automated Segmentation Tool) (Zhang et al., 2001). Global brain volumes following SIENAX analysis (Smith et al., 2002), subcortical and cortical volumes for several ROIs (Regions of Interest) were then estimated from FAST. ROIs were defined in MNI152 space, based on the Harvard-Oxford cortical and subcortical atlases (Makris et al., 2006). For this study, we identified a priori 2 ventricular, 6 bilateral subcortical and 10 bilateral cortical ROIs. We also used surface area and mean cortical thickness IDPs for two hemispheric and 9 bilateral cortical regions of interest roughly corresponding to the volumetric ROIs. Cortical surface and thickness ROIs were derived from further processing of T1 data in Freesurfer and were parcellated using the Desikan-Killiany atlas (atlas in Fig. S2) (Alfaro-Almagro et al., 2018; Desikan et al., 2006).

**Figure S2:** Desikan-Killiany parcellation, created with ggseg R package (Mowinckel and Vidal-Piñeiro, 2020).


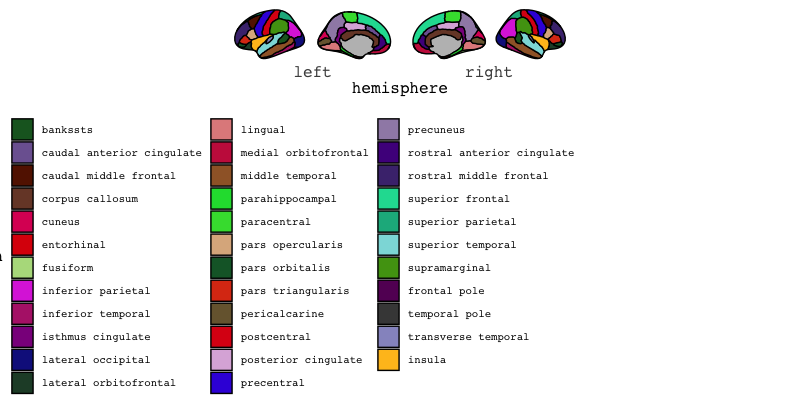

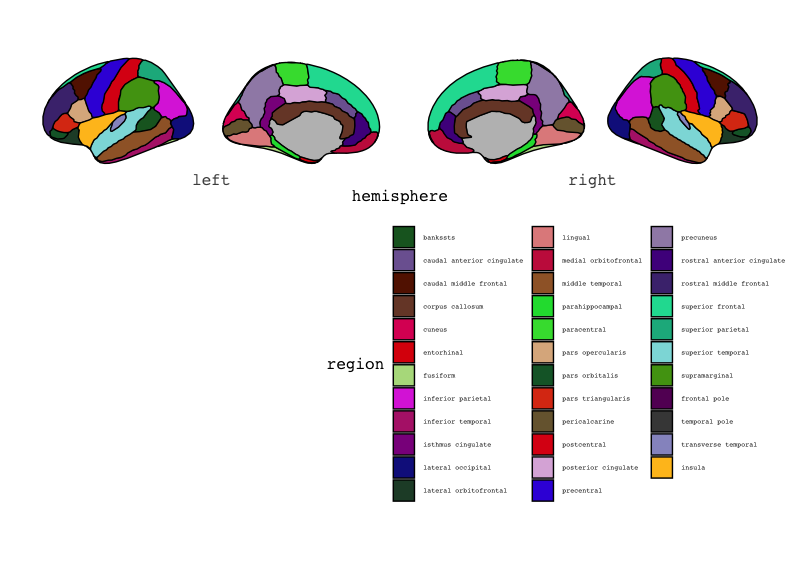


Whole brain IDPs were normalized for head size. Smaller UK Biobank ROIs were combined to form some of the ROIs used in our T1 analyses (summed for volumes and area; averaged for thickness). The lateral prefrontal cortex ROI was a composite measure of the frontal pole, superior, middle and inferior frontal gyri, according to the cortical atlases used for pre-processing and previous literature (Holmes et al., 2018). For volumes, the inferior frontal gyrus combined pars opercularis and pars triangularis; for Freesurfer measures the middle frontal gyrus combined rostral and caudal middle frontal and the inferior frontal gyrus combined the pars opercularis, pars triangularis and pars orbitalis ROIs (Harris et al., 2022).

Composite measures included also the following volume ROIs: parahippocampal gyrus (anterior and posterior division); inferior parietal cortex (supramarginal gyrus anterior division, supramarginal gyrus posterior division, angular gyrus) and, for thickness and area ROIs: anterior cingulate (caudal anterior cingulate and rostral anterior cingulate) and orbitofrontal cortex (lateral orbitofrontal cortex and medial orbitofrontal cortex (Harris et al., 2022).

### 2. Diffusion-weighted MRI (dMRI)

In the dMRI pipeline, after correcting for eddy currents, head motion and outlier-slices, and applying GDC, data were analysed in a TBSS (Tract-Based Spatial Statistics) and a probabilistic tractography pathway, both of which yielded a series of connectivity and white matter integrity measures (Alfaro-Almagro et al., 2018). We used tract measures from probabilistic tractography. The BEDPOSTx tool (Bayesian Estimation of Diffusion Parameters Obtained using Sampling Techniques) (Jbabdi et al., 2012) was used to model crossing fibres within voxels and then probtrackx/AutoPtx were run to perform tractography analysis (Behrens et al., 2007). Weighted-mean Fractional Anisotropy (FA) and weighted-mean Mean Diffusivity (MD) were estimated for 27 white matter tracts, including association, projection fibres and thalamic radiations (Cox et al., 2016):

| **Association fibres** | **Projection fibres** | **Thalamic radiations** |
| --- | --- | --- |
| Cingulate gyrus part of cingulum | Corticospinal tract | Superior thalamic radiation |
| Parahippocampal part of cingulum | Medial lemniscus | Anterior thalamic radiation |
| Superior longitudinal fasciculus | Acoustic radiation | Posterior thalamic radiation |
| Inferior longitudinal fasciculus | Major forceps |  |
| Inferior fronto-occipital fasciculus | Minor forceps |  |
| Uncinate fasciculus | Middle Cerebellar Penducle |  |

All fibres except commissural (major and minor forceps, middle cerebellar penducle) were investigated bilaterally (Fig. S3 A, B, and C, created in MRIcroGL using averaged tract maps from ~4,000 UK Biobank participants).


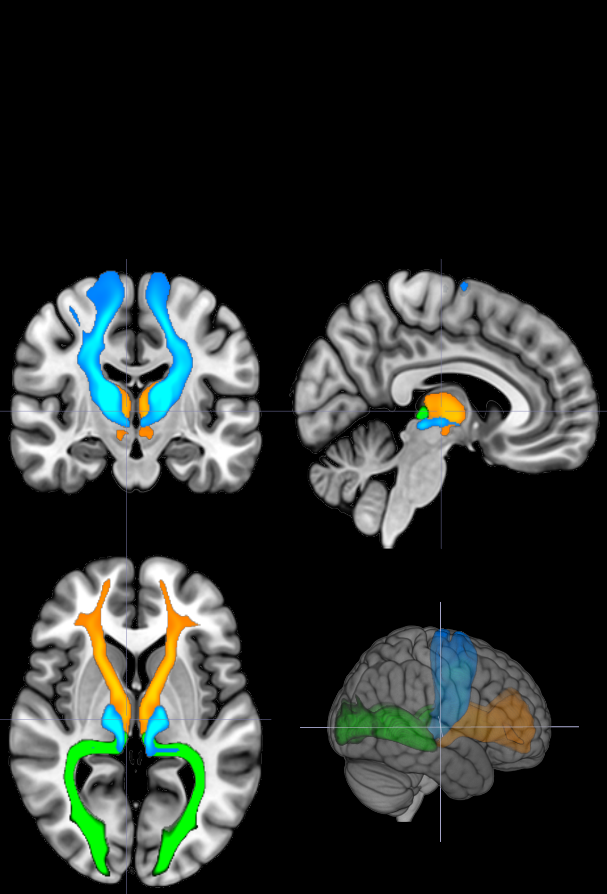


Figure S3A: Thalamic radiations

light blue: superior thalamic radiation; light orange: anterior thalamic radiation; green: posterior thalamic radiation


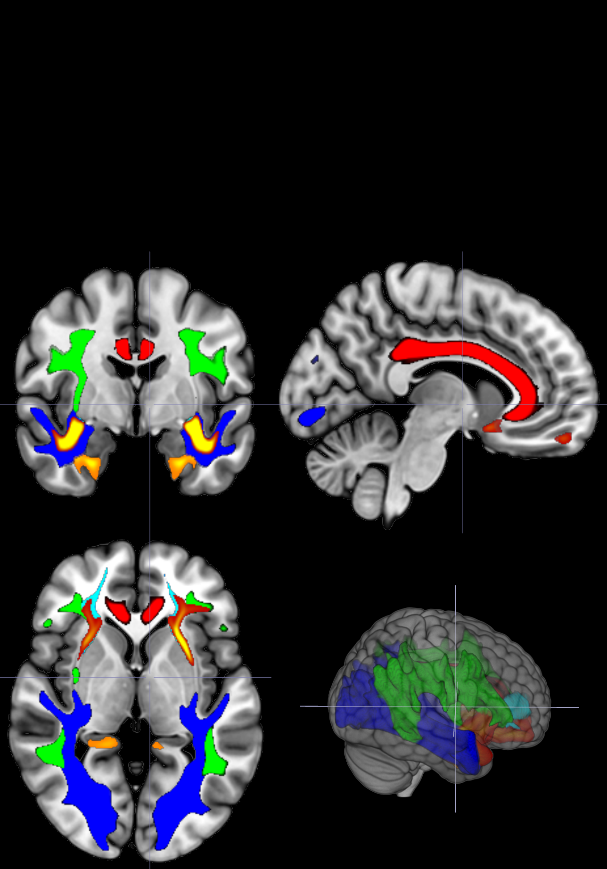


Figure S3B: Association fibres

red: cingulate gyrus part of cingulum; orange: para-hippocampal part of cingulum; green: superior longitudinal fasciculus; blue: inferior longitudinal fasciculus; light blue: inferior fronto-occipital fasciculus; yellow-red: uncinate fasciculus


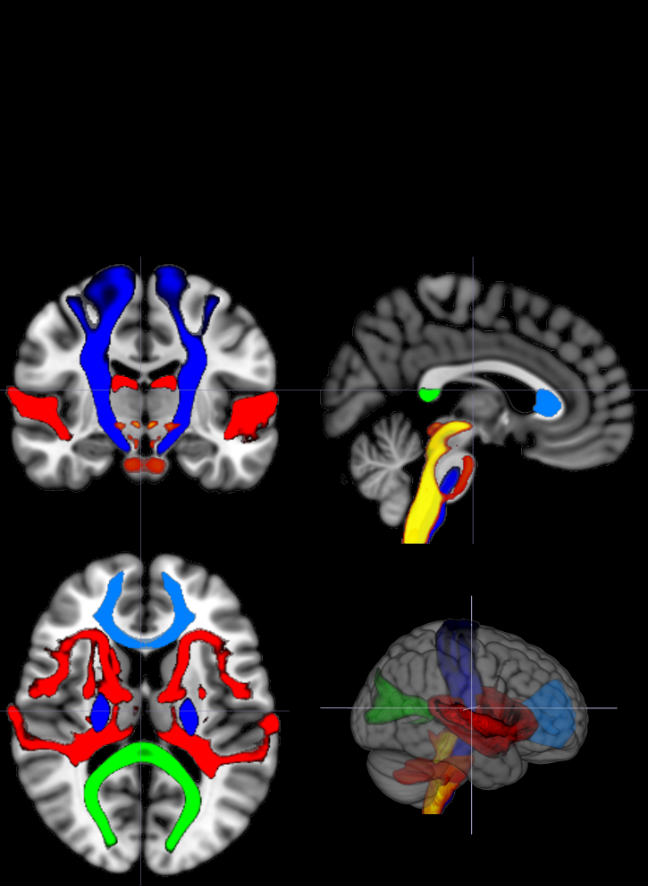


Figure S3C: Projection fibres

blue: corticospinal tract; yellow: medial lemniscus; red: acoustic radiation; green: major forceps; light blue: minor forceps; dark orange: middle cerebellar penducle

### 3. Resting-state functional MRI (rs-fMRI)

For the resting state functional MRI (rs-fMRI), initial pre-processing included motion correction, grand-mean intensity normalisation, high pass temporal filtering, echo-planar image (EPI) unwarping and GDC unwarping; structured artefacts were removed (Alfaro-Almagro et al., 2018). Using data from 4,100 UK Biobank participants, group independent component analysis (group-ICA) was performed at two dimensionalities (D=25 and D=100), each dimensionality corresponding to different number of components. Artefactual components were removed. Subject-specific node time-series were generated via dual regression and were used to derive network-matrices for each participant. The nodes of these matrices correspond to distinct “good” (not artefactual) ICA components, and the edges to connectivity strength between components. Generation of matrices was performed in FSLnets (<https://fsl.fmrib.ox.ac.uk/fsl/fslwiki/FSLNets>) using both full and partial correlation. Partial correlation was L2-regularized and Pearson coefficients were r-to-z transformed (Alfaro-Almagro et al., 2018).

Given that group-ICA with FSL MELODIC was used, resulting in non-contiguous weighted (soft-parcellation) components, for the purposes of this study we used data from high-dimensional (D=100) group ICA, where components are generally smaller and each one can be considered as a node, as opposed to low-dimensional (D=25) ICA, where edges represent connectivity between large, bulk networks of many non-contiguous spatial nodes (Miller et al., 2016). Cleaning of noise nodes by the UK Biobank resulted in a 55x55 matrix for each participant. Partial correlation matrices were used for the analysis, as these give a better estimate of direct connection between nodes than full correlation (Miller et al., 2016). The list and spatial maps for all the good nodes (n=55) can be found online (<https://www.fmrib.ox.ac.uk/ukbiobank/group_means/rfMRI_GoodComponents_d100_v1.txt>; https://www.fmrib.ox.ac.uk/ukbiobank/group_means/WINpapaya/GeneralTemplate/?path=%22../data/General/edge_list_d100.csv%22&param=0;2;Red%20Overlay;Blue%20Overlay;3;15;(91,109,91) ; accessed 20 August 2022).

## C. Statistical analysis - Inflammatory markers and Sub-analyses

Models of baseline inflammatory markers were ran among all patients with psoriasis and were fully adjusted for covariates as in the main analysis. The inflammatory markers were used as the dependent variable of interest. Models for precuneus thickness were ran both with an interaction term with depression and unadjusted for depression; and similarly the resting-state fMRI IDP model was run with and without an interaction with PsA. Only sex was included as covariate in haematology models at the time of scan, as data were available for n=36 patients. The relationships of brain IDPs with CRP were non-linear and a logarithmic term was used for CRP.

N=101 depressed participants with psoriasis had lifetime suicidality data. The model for suicidality was adjusted for age, age^2^, sex, assessment centre, intracranial volume, handedness, BMI and field 26500. For disease duration analysis, we used n=160 participants who had self-reported age of onset. This model was additionally adjusted for depression.

## D. Results - Baseline Characteristics

### 1. Clinical characteristics

**Table S4: Psoriasis characteristics**

| Characteristics | Missing data | Psoriasis, Depressed (n=131) | Psoriasis, Not depressed  (n=131) | *p*-value |
| --- | --- | --- | --- | --- |
| **Psoriasis treatment at scan** | 0.00 (0.00%) |  |  | 0.867 |
| **Systemic (conventional/ biologics)** |  | 10 (7.6%) | 9 (6.9%) |  |
| **Topical** |  | 44 (33.6%) | 48 (36.6%) |  |
| **None** |  | 77 (58.8%) | 74 (56.5%) |  |
| **Psoriasis Duration ^a^** | 34.22 (16.19%) | 32.3 (15.9) | 37.1 (16.5) | **0.059** |
| Notes: ^a^ sample of 160 participants with self-reported disease onset. | | | | |

**Table S5: Depression characteristics**

| Characteristics | Missing data | Psoriasis, Depressed (n=131) | Controls, Depressed  (n=393) | *p*-value |
| --- | --- | --- | --- | --- |
| **Longest Ever DE Duration in weeks, median (IQR)** | 78 (14.88%) | 9 (15) | 8 (20) | 0.866 |
| **Number of Lifetime DE, median (IQR)** | 106 (20.23%) | 2 (2.5) | 2 (4) | 0.173 |
| **Lifetime Treatment for Depression** | 114 (21.75%) | 67 (62.6%) | 184 (60.7%) | 0.818 |
| **Antidepressant Use at scan** | 0 (0.00%) | 4 (3.1%) | 14 (3.6%) | >0.99 |
| Notes: DE=Depressive Episode, sd=standard deviation, IQR=interquartile range | | | | |

### 2. Psoriatic Arthritis subgroup

**Table S6: Characteristics for three subgroups**

| Baseline Characteristics | Psoriasis only  n= 234 | Psoriasis + PsA n=28 | Healthy controls  n= 786 | *p*-value |
| --- | --- | --- | --- | --- |
| Sex (females) | 142 (60.7%) | 14 (50.0%) | 468 (59.5%) | 0.553 |
| Age, mean(sd) | 62.3 (7.2) | 64.5 (7.5) | 62.3 (7.3) | 0.245 |
| BMI | 26.0 (4.2) | 26.1 (5.2) | 26.1 (4.2) | 0.88 |
| Handedness (R) | 209 (89.3%) | 28 (100%) | 707 (89.9%) | 0.486 |
| Depression | 114 (48.7%) | 17 (61%) | 393 (50%) | 0.487 |
| Psoriasis treatment | | | | **<0.001** |
| Systemic (conventional/ biologics) | 9 (3.8%) | 10 (35.7%) | NA |  |
| Topical | 84 (35.9%) | 8 (28.6%) | NA |  |
| None | 141 (60.2%) | 10 (35.7%) | NA |  |
| Psoriasis Duration ^a^ | 34.22 (16.19) | 38.24 (17.57) |  | 0.296 |
| CRP, mg/L, median (IQR) ^b^ | 1.34 (1.74) | 2.17 (3.31) | 0.98 (1.69) | **<0.001** |
| Neutrophil count, 10^9 cells/L, mean (sd) ^b^ | 4.01 (1.23) | 4.11 (1.01) | 3.97 (1.29) | 0.486 |
| Neutrophil count at scan, mean (sd) | 4.17 (1.45) | 4.98 (1.37) | 4.52 (1.22) | 0.127 |
| Townsend Deprivation Index**,** median(IQR) | -2.33 (3.45) | -2.96 (3.36) | -2.57 (3.47) | 0.770 |
| Alcohol use frequency | 111 (48.1%) | 10 (35.7%) | 362 (46.2%) | 0.785 |
| Hypertension | 56 (23.7%) | 10 (35.7%) | 189 (24.1%) | 0.364 |
| Notes: PsA= Psoriatic arthritis, IQR=Interquartile range, sd=standard deviation, BMI=Body Mass Index, CRP=C-reactive protein. ^a^ sample of 160 participants with self-reported disease onset; ^b^ measured at baseline. P-Values reported for comparisons among all three groups. | | | | |

### 2. Diffusion and resting-state fMRI samples

Outcomes were missing for <5% of T1 sample in these sequences. We ran separate analyses and confirmed that the four matched groups in these sequences remained well frequency-matched for gender, age and depression phenotype (p>0.9 for between-group comparison in both modalities and all variables). The psoriasis versus non-psoriasis controls were reasonably balanced for body mass index (BMI) in both sequences (p>0.1). The PsA subgroup did not significantly differ from participants with psoriasis only and non-psoriasis controls for these variables. In the diffusion sample only (n=999), we found a difference for BMI between depressed participants without psoriasis and non-depressed participants without psoriasis (p=0.01). We have adjusted for BMI in all analyses.

## E. Results - The rs-fMRI connectome


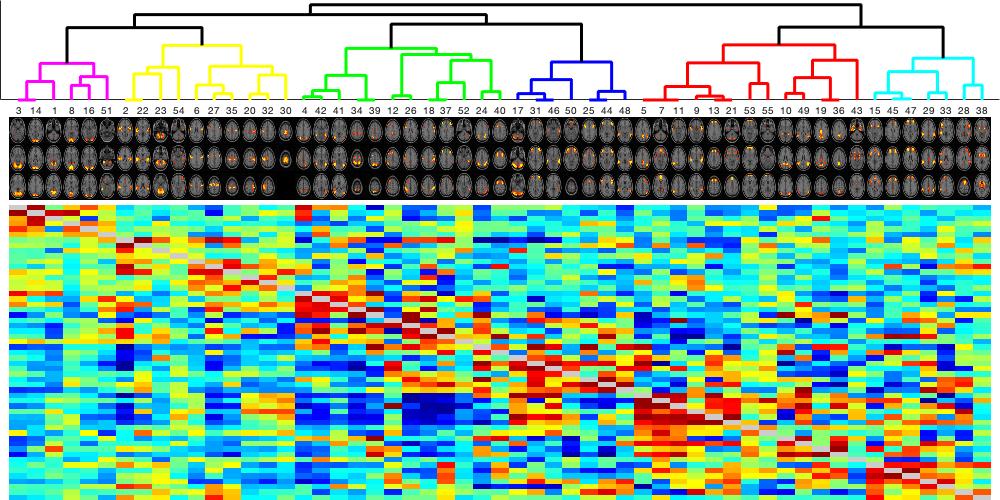

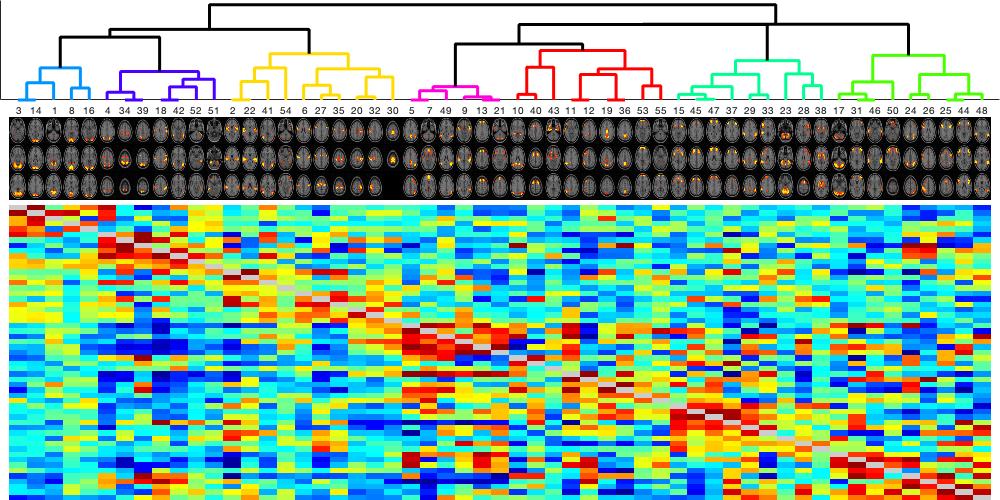

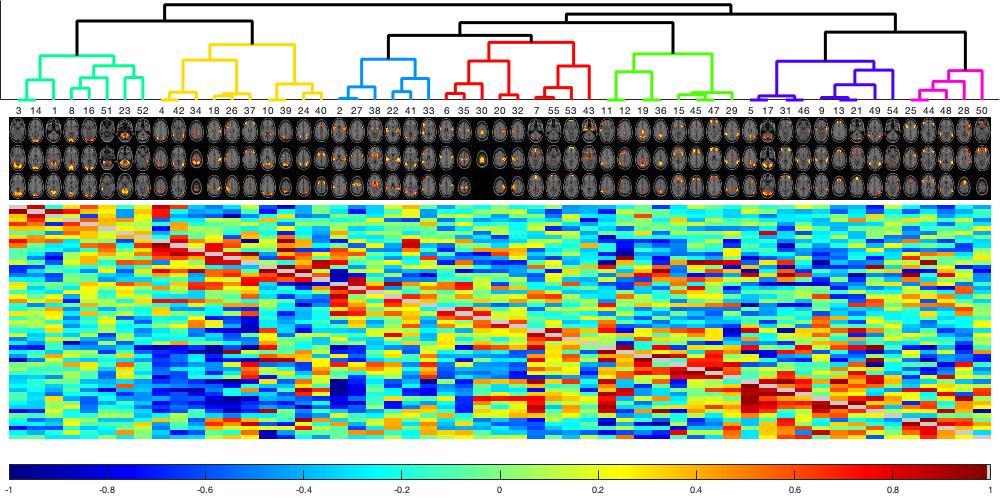


**Figure S4 (page 17):** rs-fMRI correlation matrices for controls (top), patients with psoriasis only (middle) and patients with both psoriasis and PsA (bottom). Partial correlation matrices for each group (z-transformed group-average) are represented above the diagonal; full correlation matrices for the group (z-transformed group-average) drive the hierarchical clustering (performed using Ward algorithm) and are represented below the diagonal for interpretation and visualization purposes. The group of nodes 5, 7, 9, 13, 21, 49 roughly corresponds to the Default mode network (DMN; dashed boxes); interconnectivity between these nodes was not different between groups. Noted is the gradual decoupling for psoriasis and PsA between nodes 13 and 41 (boxes pointed with arrows). Image created using FSLnets.

## F. Results - Sensitivity Analyses

After adjusting for alcohol use, Townsend deprivation index, ethnicity, education and hypertension as part of a sensitivity analysis, our results did not change in magnitude or significance (Table S7). The phenotype used to define depression did not show significant effects on the IDPs among the depressed patients. For the precuneus in particular, we re-ran the models in the total sample after we a) excluded the “Smith” depressed (n=748) and b) excluded the “CIDI” depression (n=824). Results remained significant and of similar magnitude in both cases (Effect of psoriasis x depression using only those who met CIDI criteria: Cohen’s f = 0.09; 95% CI (0.01, 0.16); using only those who met Smith criteria: Cohen’s f = 0.09; 95% CI (0.02, 0.16)).

**Table S7: Sensitivity Analysis for additional potential confounders**

| **IDP** |  | **Model 1**  **(results, pre-FDR correction)** | **Model 2**  **(additionally adjusted for alcohol, deprivation, ethnicity, education and hypertension)** |
| --- | --- | --- | --- |
| Precuneus thickness right | Interaction psoriasis x depression | Cohen’s f = 0.09; 95% CI (0.03, 0.15); p=0.0019 | Cohen’s f = 0.10; 95% CI (0.04, 0.16); p=0.0015 |
| Connectivity Nodes 13-41 | Group main effect | Cohen’s f = 0.13; 95% CI 0.06, 0.19); p<0.001 | Cohen’s f = 0.14; 95% CI (0.07, 0.20); p<0.001 |
| Notes: FDR=False Discovery Rate | | | |

## G. Additional Figures and Tables


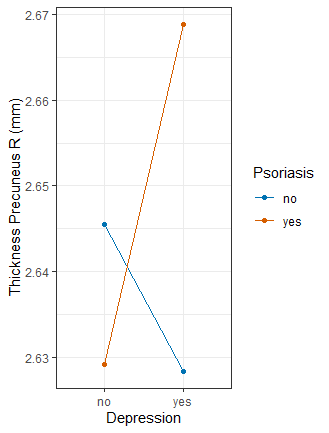


**Figure S5:** Estimated marginal means of thickness in right precuneus depending on presence of psoriasis and depression.


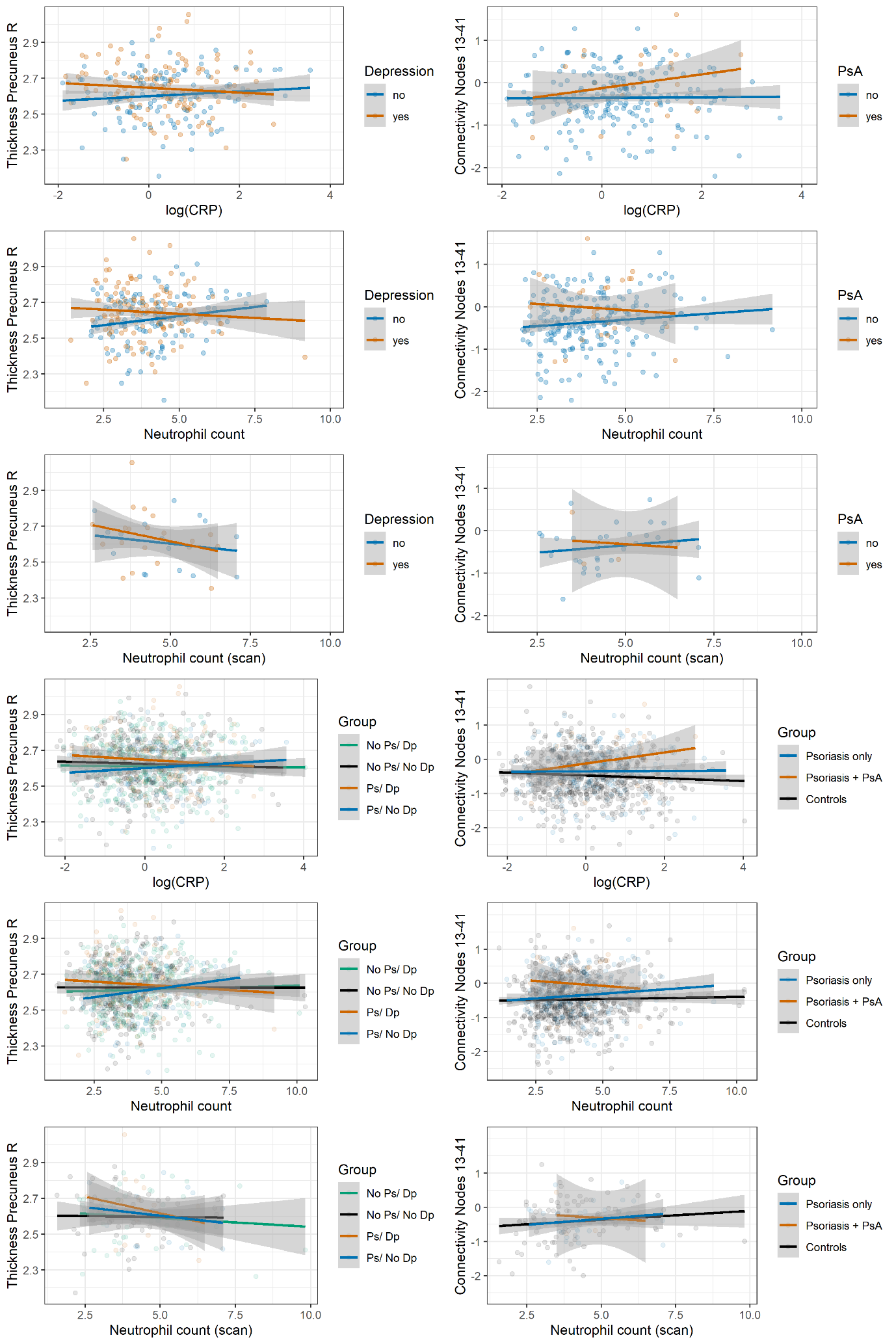


**Figure S6:** Top three rows: Relationships of brain IDPs with C-reactive protein (CRP) logarithm and neutrophil counts at baseline and at time of scan among patients with psoriasis, depending on the presence of depression and psoriatic arthritis (PsA); none of the associations were statistically significant. Precuneus thickness measured in mm; neutrophil counts measured as 10^9 cells/Litre. Connectivity measured as the z-score of the partial correlation coefficient. Bottom three rows: Diagrams presented with controls for comparative purposes. Ps=Psoriasis, Dp=Depression, PsA=Psoriatic arthritis.

**Table S8: Associations of C-reactive protein (CRP) and neutrophil counts with brain metrics of interest**

| **Investigated Biomarker** | **Dependent Variables of Interest** | **Thickness**  **Precuneus Right** | | **Connectivity Strength**  **Nodes 13-41** | |
| --- | --- | --- | --- | --- | --- |
|  |  | **Model 1** ^a^ | **Model 2** ^b^ | **Model 3** ^c^ | **Model 4** ^d^ |
| **CRP (baseline)** | CRP (baseline) | β=-0.004  (-1.34, 0.12), *p*=0.97 | β=-0.002  (-0.13, 0.13), *p*=0.97 | β=0.16  (-0.05, 0.38), *p*=0.45 | β=0.07  (-0.07, 0.20), *p*=0.68 |
|  | CRP (baseline) × Depression | β=0.09  (-2.98, 0.21), *p*=0.45 | *Not included in model* | *Not included in model* | *Not included in model* |
|  | CRP (baseline) × PsA | *Not included in model* | *Not included in model* | β=-0.14 (-0.35, 0.06), *p*=0.45 | *Not included in model* |
| **Neutrophil count (baseline)** | Neutrophil count (baseline) | β=0.003  (-1.01, 0.11), *p*=0.97 | β=-0.002  (-0.11, 0.10), *p*=0.97 | β=-0.01  (-0.22, 0.20), *p*=0.97 | β=0.06  (-0.05, 0.18), *p*=0.58 |
|  | Neutrophil count (baseline) × Depression | β=0.09  (-1.07, 0.19), *p*=0.45; *p-uncorrected*=  0.08 | *Not included in model* | *Not included in model* | *Not included in model* |
|  | Neutrophil count (baseline) × PsA | *Not included in model* | *Not included in model* | β=0.08  (-0.13, 0.29), *p*=0.78 | *Not included in model* |
| Notes: All results as standardised β (95% confidence intervals), Benjamini-Hochberg adjusted p-value. All uncorrected p-values were > 0.1, except where otherwise specified.  The table reports biomarkers at baseline. Models which were only sex-adjusted were run for neutrophil counts at the time of scan; the correlation coefficient was β=-0.20 (-0.50, 1.35), p=0.45; p-uncorrected= 0.09 for precuneus thickness; β=-0.03 (-0.65, 1.35), p=0.97 for node 13-41 connectivity strength.  ^a^ Fully adjusted for confounders, including depression × biomarker term. ^b^ Not adjusted for depression or depression × biomarker interaction. ^c^ Fully adjusted for confounders, including PsA (Psoriatic arthritis) × biomarker term. ^d^ Not adjusted for PsA or PsA × biomarker interaction. | | | | | |
|  | | | | | |

**Table S9:** **Secondary Analysis: Associations of thrichotomized group (psoriasis, psoriatic arthritis, controls) and depression (presence/absence) with Mean Diffusivity (MD)**

| ROIs | Thrichotomized Group | | | | Depression | | | | | Interaction Group *Depression | | | | |
| --- | --- | --- | --- | --- | --- | --- | --- | --- | --- | --- | --- | --- | --- | --- |
|  | p | *BH*-p | F | ω^2^ | | p | *BH*-p | F | ω^2^ | | p | *BH*-p | F | ω^2^ |
| PCA-derived MD | | | | | | | | | | | | | | |
| global | 0.619 | 0.826 | 0.480 | -0.001 | | 0.128 | 0.194 | 2.315 | 0.002 | | **0.023** | **0.038** | 3.786 | 0.003 |
| association fibres | 0.593 | 0.826 | 0.523 | -0.001 | | 0.184 | 0.194 | 1.768 | 0.001 | | **0.028** | **0.038** | 3.581 | 0.004 |
| projection fibres | 0.946 | 0.946 | 0.055 | -0.002 | | 0.194 | 0.194 | 1.690 | 0.001 | | 0.170 | 0.170 | 1.778 | 0.001 |
| thalamic radiations | 0.512 | 0.826 | 0.670 | 0.000 | | 0.100 | 0.194 | 2.719 | 0.001 | | **0.022** | **0.038** | 3.824 | 0.004 |
| LEFT |  |  |  |  | |  |  |  |  | |  |  |  |  |
| acoustic radiation | 0.814 | 0.895 | 0.206 | -0.002 | | 0.350 | 0.506 | 0.875 | 0.000 | | 0.700 | 0.700 | 0.356 | -0.001 |
| anterior thalamic radiation | 0.229 | 0.895 | 1.478 | 0.001 | | 0.658 | 0.751 | 0.196 | -0.001 | | 0.102 | 0.177 | 2.292 | 0.002 |
| cingulate gyrus  part of cingulum | 0.522 | 0.895 | 0.650 | -0.001 | | 0.086 | 0.255 | 2.961 | 0.002 | | **0.013** | 0.072 | 4.353 | 0.000 |
| corticospinal tract | 0.513 | 0.895 | 0.669 | -0.001 | | 0.064 | 0.255 | 3.426 | 0.002 | | 0.067 | 0.140 | 2.704 | 0.003 |
| inferior fronto-  occipital fasciculus | 0.607 | 0.895 | 0.500 | -0.001 | | 0.170 | 0.382 | 1.888 | 0.001 | | **0.046** | 0.140 | 3.090 | 0.003 |
| inferior longitudinal fasciculus | 0.862 | 0.895 | 0.148 | -0.001 | | 0.244 | 0.440 | 1.357 | 0.000 | | 0.105 | 0.177 | 2.259 | 0.002 |
| medial lemniscus | 0.469 | 0.895 | 0.759 | 0.000 | | 0.312 | 0.495 | 1.025 | 0.000 | | 0.157 | 0.223 | 1.854 | 0.002 |
| parahippo-campal  part of cingulum | 0.727 | 0.895 | 0.319 | -0.001 | | **0.038** | 0.255 | 4.330 | 0.003 | | 0.056 | 0.140 | 2.887 | 0.003 |
| posterior thalamic radiation | 0.947 | 0.947 | 0.054 | -0.002 | | 0.305 | 0.495 | 1.052 | 0.000 | | 0.529 | 0.621 | 0.637 | -0.001 |
| superior longitudinal fasciculus | 0.858 | 0.895 | 0.153 | -0.001 | | 0.469 | 0.576 | 0.524 | 0.000 | | 0.094 | 0.177 | 2.368 | 0.002 |
| superior thalamic radiation | 0.735 | 0.895 | 0.308 | -0.001 | | 0.058 | 0.255 | 3.589 | 0.002 | | **0.007** | 0.072 | 5.052 | 0.006 |
| uncinate fasciculus | 0.426 | 0.895 | 0.853 | 0.000 | | 0.446 | 0.574 | 0.581 | 0.000 | | 0.062 | 0.140 | 2.785 | 0.003 |
| RIGHT |  |  |  |  | |  |  |  |  | |  |  |  |  |
| acoustic radiation | 0.432 | 0.895 | 0.839 | 0.000 | | 0.132 | 0.325 | 2.268 | 0.001 | | 0.145 | 0.218 | 1.935 | 0.002 |
| anterior thalamic radiation | 0.069 | 0.895 | 2.683 | 0.002 | | 0.243 | 0.440 | 1.364 | 0.000 | | 0.060 | 0.140 | 2.830 | 0.003 |
| cingulate gyrus  part of cingulum | 0.426 | 0.895 | 0.854 | -0.001 | | **0.008** | 0.216 | 7.060 | 0.001 | | **0.003** | 0.072 | 5.947 | 0.004 |
| corticospinal tract | 0.765 | 0.895 | 0.268 | -0.001 | | 0.227 | 0.440 | 1.462 | 0.000 | | 0.249 | 0.320 | 1.234 | 0.000 |
| inferior fronto-  occipital fasciculus | 0.667 | 0.895 | 0.405 | -0.001 | | 0.089 | 0.255 | 2.889 | 0.002 | | **0.019** | 0.072 | 3.999 | 0.005 |
| inferior longitudinal fasciculus | 0.818 | 0.895 | 0.200 | -0.001 | | 0.094 | 0.255 | 2.816 | 0.001 | | 0.066 | 0.140 | 2.722 | 0.003 |
| medial lemniscus | 0.268 | 0.895 | 1.319 | 0.001 | | 0.926 | 0.926 | 0.009 | -0.001 | | 0.575 | 0.646 | 0.554 | -0.001 |
| parahippo-campal  part of cingulum | 0.803 | 0.895 | 0.220 | -0.001 | | 0.409 | 0.553 | 0.681 | 0.000 | | 0.190 | 0.257 | 1.663 | 0.001 |
| posterior thalamic radiation | 0.798 | 0.895 | 0.225 | -0.001 | | 0.059 | 0.255 | 3.572 | 0.002 | | 0.138 | 0.218 | 1.982 | 0.002 |
| superior longitudinal fasciculus | 0.375 | 0.895 | 0.982 | 0.000 | | 0.094 | 0.255 | 2.803 | 0.002 | | **0.008** | 0.072 | 4.482 | 0.006 |
| superior thalamic radiation | 0.740 | 0.895 | 0.301 | -0.001 | | 0.076 | 0.255 | 3.161 | 0.002 | | **0.014** | 0.072 | 4.294 | 0.005 |
| uncinate fasciculus | 0.360 | 0.895 | 1.022 | 0.000 | | 0.922 | 0.926 | 0.010 | -0.001 | | 0.295 | 0.363 | 1.221 | 0.000 |
| COMMISSURAL | | | | | | | | | | | | | | |
| forceps major | 0.534 | 0.895 | 0.628 | -0.001 | | 0.667 | 0.751 | 0.185 | -0.001 | | 0.636 | 0.661 | 0.452 | -0.001 |
| forceps minor | 0.598 | 0.895 | 0.514 | -0.001 | | 0.356 | 0.506 | 0.852 | 0.000 | | **0.017** | 0.072 | 4.111 | 0.005 |
| middle cerebellar peduncle | 0.319 | 0.895 | 1.144 | 0.000 | | 0.731 | 0.790 | 0.118 | -0.001 | | 0.634 | 0.661 | 0.455 | -0.001 |
| Notes: BH-p: Benjamini-Hochberg adjusted p-value | | | | | | | | | | | | | |  |

**References**

Alfaro-Almagro, F., et al., 2018. Image processing and Quality Control for the first 10,000 brain imaging datasets from UK Biobank. Neuroimage 166, 400-424.

Behrens, T.E., et al., 2007. Probabilistic diffusion tractography with multiple fibre orientations: What can we gain? Neuroimage 34, 144-155.

Cox, S.R., et al., 2016. Ageing and brain white matter structure in 3,513 UK Biobank participants. Nat. Commun. 7, 13629

Davis, K.A., et al., 2020. Mental health in UK Biobank–development, implementation and results from an online questionnaire completed by 157 366 participants: a reanalysis. BJPsych open 6.

Desikan, R.S., et al., 2006. An automated labeling system for subdividing the human cerebral cortex on MRI scans into gyral based regions of interest. Neuroimage 31, 968-980.

Glanville, K.P., et al., 2021a. Multiple measures of depression to enhance validity of major depressive disorder in the UK Biobank. BJPsych open 7.

Glanville, K.P., et al., 2021b. Investigating pleiotropy between depression and autoimmune diseases using the UK Biobank. Biological psychiatry global open science 1, 48-58.

Harris, M.A., et al., 2022. Structural neuroimaging measures and lifetime depression across levels of phenotyping in UK biobank. Transl. Psychiatry 12, 1-9.

Holmes, S.E., et al., 2018. Elevated translocator protein in anterior cingulate in major depression and a role for inflammation in suicidal thinking: a positron emission tomography study. Biol. Psychiatry 83, 61-69.

Jbabdi, S., et al., 2012. Model‐based analysis of multishell diffusion MR data for tractography: How to get over fitting problems. Magn. Reson. Med. 68, 1846-1855.

Jenkinson, M., et al., 2005. BET2: MR-based estimation of brain, skull and scalp surfaces. Eleventh annual meeting of the organization for human brain mapping. Toronto., p. 167.

Makris, N., et al., 2006. Decreased volume of left and total anterior insular lobule in schizophrenia. Schizophr. Res. 83, 155-171.

Miller, K.L., et al., 2016. Multimodal population brain imaging in the UK Biobank prospective epidemiological study. Nat. Neurosci. 19, 1523-1536.

Mowinckel, A.M., Vidal-Piñeiro, D., 2020. Visualization of brain statistics with R packages ggseg and ggseg3d. Advances in Methods and Practices in Psychological Science 3, 466-483.

Smith, D.J., et al., 2013. Prevalence and characteristics of probable major depression and bipolar disorder within UK biobank: cross-sectional study of 172,751 participants. PLoS One 8, e75362.

Smith, S.M., et al., 2002. Accurate, robust, and automated longitudinal and cross-sectional brain change analysis. Neuroimage 17, 479-489.

Zhang, Y., et al., 2001. Segmentation of brain MR images through a hidden Markov random field model and the expectation-maximization algorithm. IEEE Trans. Med. Imaging 20, 45-57.
